# Robust stochastic Turing patterns in the development of a one-dimensional cyanobacterial organism

**DOI:** 10.1371/journal.pbio.2004877

**Published:** 2018-05-04

**Authors:** Francesca Di Patti, Laura Lavacchi, Rinat Arbel-Goren, Leora Schein-Lubomirsky, Duccio Fanelli, Joel Stavans

**Affiliations:** 1 Università degli Studi di Firenze, Dipartimento di Fisica e Astronomia, Sesto Fiorentino, Italia; 2 CSDC and INFN Sez.di Firenze, Sesto Fiorentino, Italia; 3 Department of Physics of Complex Systems, Weizmann Institute of Science, Rehovot, Israel; Massachusetts Institute of Technology, United States of America

## Abstract

Under nitrogen deprivation, the one-dimensional cyanobacterial organism *Anabaena* sp. PCC 7120 develops patterns of single, nitrogen-fixing cells separated by nearly regular intervals of photosynthetic vegetative cells. We study a minimal, stochastic model of developmental patterns in *Anabaena* that includes a nondiffusing activator, two diffusing inhibitor morphogens, demographic fluctuations in the number of morphogen molecules, and filament growth. By tracking developing filaments, we provide experimental evidence for different spatiotemporal roles of the two inhibitors during pattern maintenance and for small molecular copy numbers, justifying a stochastic approach. In the deterministic limit, the model yields Turing patterns within a region of parameter space that shrinks markedly as the inhibitor diffusivities become equal. Transient, noise-driven, stochastic Turing patterns are produced outside this region, which can then be fixed by downstream genetic commitment pathways, dramatically enhancing the robustness of pattern formation, also in the biologically relevant situation in which the inhibitors' diffusivities may be comparable.

## Author summary

Multicellular organisms, from simple to complex, often undergo a developmental process in which cells differentiate into various types, improving survivability under adverse conditions. We study experimentally and theoretically the developmental mechanism of pattern formation in *Anabaena* sp. PCC 7120, a multicellular cyanobacterial organism of ancient origin, which forms one-dimensional patterns of single, nitrogen-fixing cells separated by nearly regular intervals of photosynthetic vegetative cells, under nitrogen-poor conditions. By following the developmental process at the level of single cells in real time, we show directly that two genes involved in the inhibition of a nondiffusing activator have different spatiotemporal roles and discuss why a classical, deterministic Turing mechanism may not describe pattern formation in this system. Our stochastic model, which incorporates inevitable fluctuations in molecular numbers or demographic noise, suggests a much more robust mechanism of pattern formation: Noise can seed the formation of transient, stochastic Turing patterns for parameter values in which deterministic patterns do not form. These patterns can then be fixed by downstream genetic commitment pathways. This robust scenario of pattern formation may apply to a wide range of developmental pattern-forming systems.

## Introduction

The emergence of multicellularity, together with cell differentiation and the ensuing division of labor, conferred unique advantages to the survival of organisms and paved the way for the evolution of patterned complex forms such as those extant today. Among the remarkable diversity of organismal shapes, nearly periodic structures such as digits in a limb [1], sensory bristles in *Drosophila* [2], palatal ridges [3], and stripes in zebrafish [4] represent a fundamental and ubiquitous motif, suggesting that common mechanisms may be at play behind these structures’ morphogenesis. A striking example of nearly periodic developmental patterns is displayed by cyanobacterial *Anabaena* sp. PCC 7120 filaments (henceforth *Anabaena*) [5, 6]. In nitrogen-rich environments, *Anabaena* exhibits undifferentiated filaments of vegetative cells that carry out both oxygenic photosynthesis and assimilation of combined nitrogen sources such as ammonium or nitrates. However, when combined nitrogen sources become scarce, *Anabaena* can fix atmospheric nitrogen using nitrogenase, an enzyme whose function is abolished by minute amounts of oxygen. Thus, photosynthesis and nitrogen fixation are incompatible processes within the same cell, an incompatibility that the organism solves by the differentiation of some of its cells into heterocysts, cells that specialize in nitrogen fixation but carry out no oxygenic photosynthesis. Heterocysts contain an extra cell envelope relative to their vegetative counterparts. This cell envelope is comprised of two different layers, one made of glycolipids and the other of polysaccharide. The glycolipid layer appears to have a reduced permeability to gases, allowing heterocysts to maintain a micro-oxic environment [7]. A developmental pattern of individual heterocysts separated by nearly regular intervals of about 10–15 vegetative cells forms, with heterocysts supplying surrounding vegetative cells with fixed nitrogen products while receiving carbohydrate products from their neighbors in return. This characteristic lengthscale is independent of filament length. Since heterocysts lose the ability to divide, well-developed filaments grow by the growth and division of vegetative cells. When a vegetative cell interval becomes long enough, a new intercalary heterocyst forms in its midst, thereby maintaining the characteristic lengthscale of the developmental pattern. This organization represents one of the earliest experiments of differentiated multicellularity on Earth and can be traced back to more than 2 billion years ago [7].

The developmental cascade giving rise to de novo pattern formation from undifferentiated filaments is triggered upon nitrogen step-down by the concerted action of the NtcA and HetR protein regulators [8]. NtcA is activated by binding of 2-oxoglutarate, which accumulates in cyanobacteria under nitrogen deprivation [7]. HetR regulates itself through a positive feedback loop that not only amplifies its mean levels [9, 10] but also enhances variations between cells or noise [11]. Levels of HetR grow in clusters of contiguous cells, but only one cell eventually commits fully to differentiation into a heterocyst, while the others revert into a regular, vegetative state. Commitment into a heterocyst state, which is irreversible, is mediated by the HetP protein [12]. Resolution of clusters is achieved by lateral inhibition effected by PatS, whose production is induced by HetR early after nitrogen step-down. The gene *patS* encodes a short peptide whose C-terminal domain is post-transcriptionally processed to yield the hepta-peptide PatS-7 with the sequence RGSGR, which is believed to diffuse to neighboring cells [13, 14]. There, it interferes with the DNA-binding activity of HetR and causes its degradation, creating HetR gradients along filaments [15]. Immunity of HetR against PatS inhibition within the same cell has been proposed to be mediated by functions of the HetC and PatA proteins [10, 13, 16–18]. In contrast to PatS, production of the HetN protein takes place later during the differentiation process, and a HetN-derived signal produced predominantly at heterocysts [19–21] is also thought to diffuse between cells and inhibit HetR function there by mediating its post-translational decay. While HetN carries a hexapeptide ERGSGR in its sequence that is necessary for its inhibitory function [20, 21], neither the precise identity nor the mechanism of action of the actual HetN-derived signal is known. Overexpression of either PatS or HetN leads to complete suppression of heterocyst formation, whereas HetR overexpression can cause heterocysts to form, even under nitrogen-replete conditions [10].

**Fig 1:**
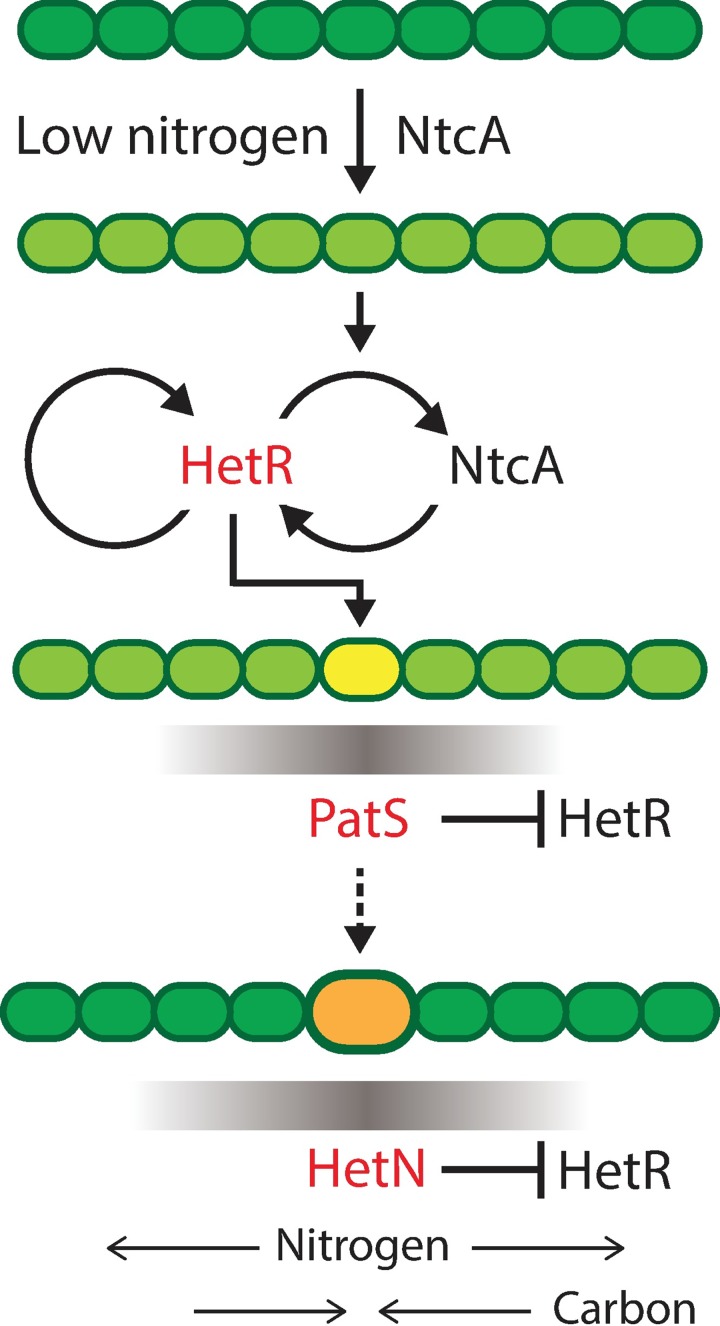
Basic regulatory network leading to heterocyst differentiation. In response to combined nitrogen deprivation, the NtcA protein is activated, leading to activation of expression of HetR in some cells. NtcA and HetR undergo mutual amplification, resulting in increased levels of the two regulators. In addition, HetR positively regulates its own production. During the early stages of differentiation, HetR induces expression of PatS in cells that can potentially form heterocysts (yellow). A PatS-derived peptide signal is thought to diffuse to neighbouring cells (grey gradient), where it interferes with the DNA-binding activity of HetR, causing its degradation and creating HetR gradients along filaments. At late stages (dashed arrow), HetN is produced in heterocysts (orange), and a HetN-derived signal is conveyed to neighbouring cells (grey gradient), where it inhibits HetR function and heterocyst formation. During the differentiation process, cells lose phycobilisomes and their autofluorescence declines (different shades of green). Phycobilisomes are restored when nitrogen compounds produced by the heterocysts reach the vegetative cells. For details, see Introduction. The dynamical variables of our model, the activator HetR and its inhibitors PatS and HetN, have been emphasized in red. Adapted with modifications from [6].

The presence of activator and inhibitor species, cell–cell communication, an intrinsic lengthscale of patterns that is independent of filament length, and the de novo formation of patterns from a homogeneous state has suggested that a diffusion-driven Turing mechanism may be behind pattern formation in *Anabaena* [22]. In Turing’ s classic model of morphogenesis, two mutually interacting substances termed “morphogens” can diffuse within a continuum domain of fixed size. Nonhomogeneous patterns can arise from a homogeneous state, provided that one of the morphogens activates the production of the other, while the latter inhibits production of the former by feedback, and when the diffusivity of the inhibitor greatly exceeds that of the activator. In spite of various commonalities, there are substantial differences between the classic, two-component Turing model and pattern formation in *Anabaena*. The Turing instability requires diffusion of both activator and inhibitor species and a large difference in the two diffusivities. Lack of diffusion of one prevents pattern formation [23], and a spatially homogeneous state remains stable. However, diffusion-driven Turing instabilities can arise in three-component models in which one of the species does not diffuse [24, 25]. There is no evidence for diffusion of the activator HetR between cells along *Anabaena* filaments, and there are two HetR inhibitors instead of one: PatS and HetN. Moreover, the diffusion constants of PatS- and HetN-derived morphogens may be comparable. The equations for the Turing model are defined on a continuous spatial support of fixed size, whereas *Anabaena* filaments continually grow by cell growth and division. *Anabaena* patterns are intrinsically discrete, with a typical lengthscale of the order of 10 cells, far from any continuum approximation [26]. A schematic layout of the basic regulatory network leading to heterocyst differentiation is displayed in Fig 1.

Upon nitrogen step-down, patterns in *Anabaena* readily form, displaying a large degree of robustness and plasticity to variations in external conditions. For example, the fraction of heterocyst cells changes in response to illumination levels [27, 28] and exogenous fixed-nitrogen levels [29, 30]. This stands in stark contrast with the exquisite dependence of Turing patterns on initial conditions, their appearance only in a small region of parameter space [31], and the large difference in the diffusivities of the activator and inhibitor morphogens that is required for the homogeneous state to be unstable. This so-called fine-tuning problem can be largely overcome and robustness enhanced if copy number fluctuations (also called demographic noise), as stemming from, e.g., gene expression noise, are significant, seeding the formation of stochastic Turing patterns in regions of parameter space where a homogeneous state is linearly stable [32–39]. Remarkably, these fluctuation-driven patterns can appear even when the diffusion constants of the activator and inhibitors are of similar magnitude and when only one species undergoes diffusion [40]. In general, the amplitude of fluctuation-driven patterns scales with the strength of the driving noise [41]. Giant amplification, however, can be produced by the interplay between noise and nonorthogonal eigenvectors of the linear stability matrix [42].

In this work, we present a model of developmental pattern formation in *Anabaena* that includes the HetR activator and its two inhibitors, PatS and HetN, as the three dynamical variables. We motivate this choice of variables by presenting evidence for the different spatiotemporal roles that PatS and HetN play during pattern maintenance. Furthermore, we incorporate demographic noise in a stochastic formulation of the model and explore the consequences of filament growth on pattern formation. We demonstrate that noise-driven, stochastic Turing patterns of sufficiently large amplitude (proportional to the strength of finite size fluctuations) to trigger commitment to differentiation can serve as a robust basis to describe developmental patterns in *Anabaena*.

## The stochastic model

### Choice of dynamical variables

The large variability in the choice of dynamical variables in previous theoretical studies highlights a lack of consensus as to what ingredients a minimal model should include to capture the essential features of development in *Anabaena* [43–48]. These studies have considered different combinations among HetR, NtcA, fixed nitrogen, HetN, and PatS. By and large, most models have been guided by the prevailing view that the functions of PatS and HetN inhibitors of HetR are well separated in time, with PatS acting during de novo pattern formation and HetN during pattern maintenance (e.g., [49]). A notable exception is the work by Zhu and colleagues [48], who included both PatS and HetN during pattern maintenance. Here, we provide experimental evidence that sheds light on the different spatiotemporal roles that both PatS and HetN play during pattern maintenance, supporting the choice of HetR, PatS, and HetN as dynamical variables. To do so, we use the enhanced decline in autofluorescence due to photosynthetic activity in a proheterocyst compared to vegetative cells as a temporal reference point. This decline takes place following nitrogen deprivation due to the degradation of phycobilisome antennae, which are the main light-harvesting complexes in cyanobacteria [50].

**Fig 2:**
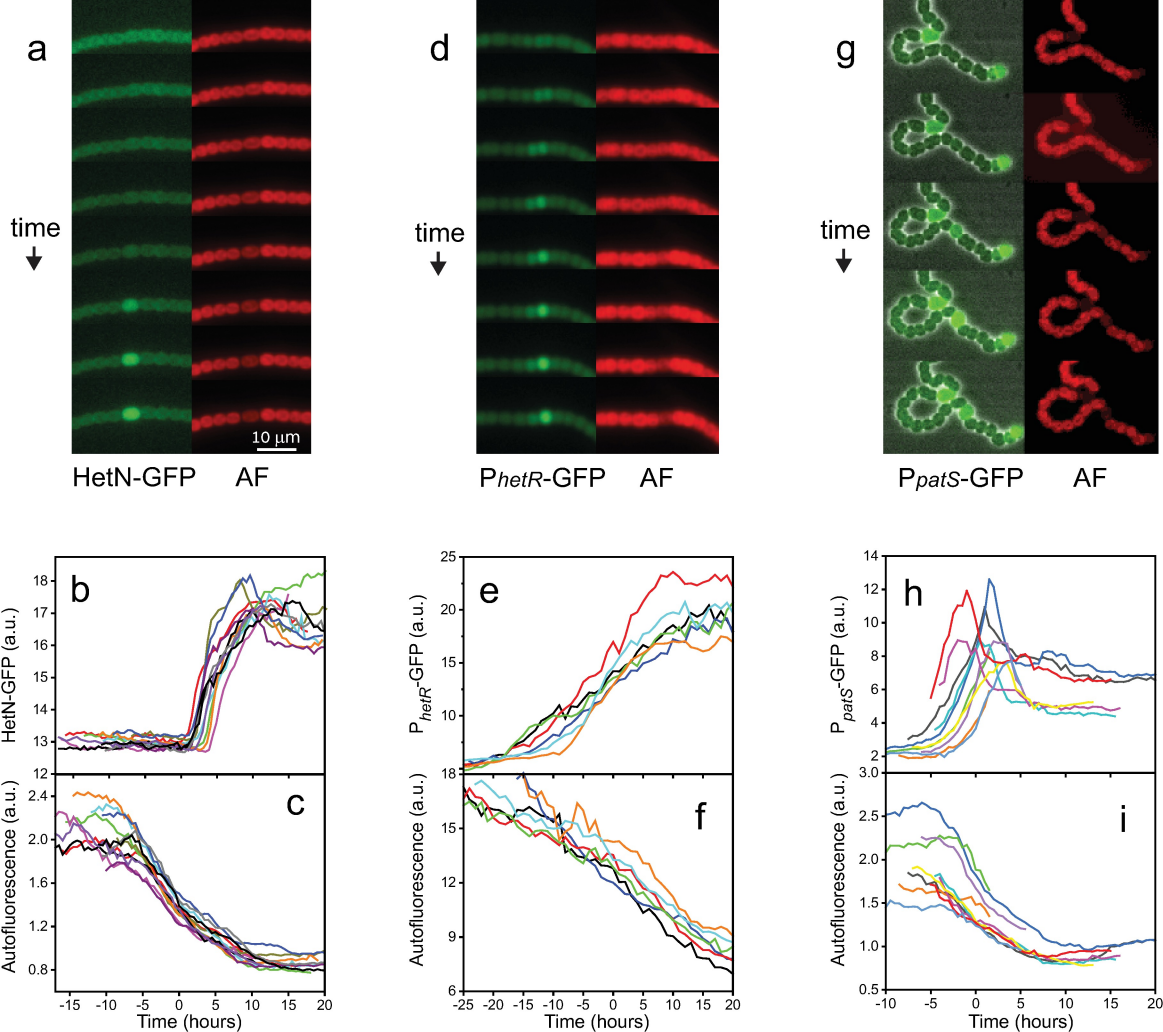
PatS and HetN have different spatiotemporal roles during pattern maintenance. Filaments under nitrogen deprivation conditions on a gel pad were followed over time as they developed (see Methods). (**a**) Expression of HetN-GFP during the formation of an intercalary heterocyst (left panels) and decline in AF from photosynthetic pigments (right panels). The interval between adjacent snapshots is 90 min. (**b,c**) Traces of fluorescence and AF from events as in (a). (**d**) Snapshots of *PhetR*-GFP expression (left panels) and AF (right panels) in the central portion of a vegetative interval between two heterocysts. The interval between adjacent snapshots is 90 min. (**e,f**) Traces of fluorescence and AF as a function of time from events as in (d). (**g**) Expression from *PpatS-gfp* overlaid on phase contrast images (left panels) and decline in photosynthetic AF (right panels) during the formation of an intercalary heterocyst. The interval between these snapshots is 2.5 h. (**h,i**) Traces of fluorescence and AF from events as in (g). The scale bar in (a) is the same for (d) and (g). The traces in (b,c), (e,f), and (h,i) have been displaced along the temporal axis so that the time corresponding to half the AF decay in all cells (defining *t* = 0) coincides. The data used in this figure are included in S1 Data. AF, autofluorescence.

An increase in fluorescence in an individual cell along a filament bearing a chromosomal *hetN-gfp* fusion is illustrated in the left snapshots in Fig 2A, together with corresponding snapshots of photosynthetic autofluorescence on the right. Significant production of HetN-GFP (green fluorescent protein) clearly takes place after the onset of decline in photosynthetic activity in the same cell. To quantify this delay, we plot in Fig 2B and 2C the fluorescence of HetN-GFP and autofluorescence, both normalized by the cell area, for a number of cells displaying behavior similar to that in Fig 2A. The traces of both autofluorescence and HetN-GFP fluorescence density of each cell have been shifted in time by the same amount so that the autofluorescence traces of all cells coincide at the midpoint of their decay. There are two salient features in these figures. First, all HetN-GFP traces collapse by this shift, demonstrating the high temporal precision of the developmental program; second, activation of HetN-GFP production is highly switchlike. Together, these features show that, under our experimental conditions, the onset of a significant decline in autofluorescence precedes HetN production by about 5 h. Note that there is a small decline in photosynthetic activity in all cells after nitrogen deprivation [51].

The increase in *hetR* expression in a pair of contiguous cells near the middle region of a vegetative interval between mature heterocysts, and the ensuing lateral inhibition that results in a unique incipient intercalary heterocyst is illustrated in the left panels in Fig 2D. The series of snapshots, taken at 90 min intervals, show the change in the expression of a chromosomally-encoded *PhetR-gfp* fusion in a cluster of vegetative cells. Shown in the panels to the right are a corresponding set of snapshots, illustrating the decline in photosynthetic autofluorescence in the cell that eventually exhibits higher fluorescence from *PhetR-gfp* expression and in which a new heterocyst will form. The snapshots show that the onset of expression from *PhetR-gfp* and the lateral inhibition of the neighbor cell clearly precede the onset of decline in photosynthetic activity. This is quantified in Fig 2E and 2F, where we show traces of fluorescence from *PhetR-gfp* and autofluorescence of cells that will become heterocysts, respectively, both normalized by cell area. The traces of both *PhetR-gfp* fluorescence and autofluorescence were shifted in time by the same amount as in the case of HetN-GFP (Fig 2B and 2C). Since significant HetN production takes place well after the onset of reduction in autofluorescence, HetN cannot mediate the lateral inhibition that resolves clusters of cells with higher expression of *PhetR-gfp* into one new intercalary heterocyst. Thus, we posit that this lateral inhibition must be mediated by PatS, a role that is identical to the one it has during de novo pattern formation. Lastly, we demonstrate PatS production during pattern maintenance directly in the series of snapshots in Fig 2G taken at 2.5-h intervals. Shown is a filament expressing *PpatS-gfp* 33 h after nitrogen step-down. The filament displays two heterocysts separated by a vegetative interval. At later times, a new intercalary heterocyst forms within this interval, with clear production from the *patS* promoter. Quantification of events such as those in (g) is shown in Fig 2H–2I. Corresponding traces in both (h) and (i) have been displaced along the time axis by the same amount as in Fig 2B and 2C. Note that the onset of expression from *PpatS-gfp* takes place slightly before or at the onset in the decline in autofluorescence, consistently with the role we posited for PatS in mediating lateral inhibition during incipient intercalary heterocyst formation. Together, the above data support the notion that HetN and PatS are both necessary for pattern maintenance and lead us to choose HetR, PatS, and HetN as dynamical variables in our model.

### Demographic noise levels in *Anabaena* are significant

In order to justify a stochastic approach to model development in *Anabaena* and the use of master equations to describe the dynamics of the HetR, PatS, and HetN, it is important to show that copy numbers of regulators are typically small, and thus, number fluctuations lead to a high level of demographic noise. We present evidence that this is so in the case of production from the *hetR* promoter. We calibrated fluorescence measurements in terms of absolute copy numbers of GFP molecules *n*_*GFP*_ from a *PhetR-gfp* fusion, exploiting the statistics of binary partitions of proteins between a cell and its daughters following cell division [52]. These measurements were carried out in filaments with a wild-type background (see Methods). We obtained *n*_*GFP*_ = 41 ± 17 for a typical cell under nitrogen-replete conditions and *n*_*GFP*_ = 40 ± 14 for a vegetative cell between two heterocysts under nitrogen-poor conditions. Note that these numbers actually represent an upper bound on HetR numbers, since the lifetime of GFP is of many hours and GFP does not report on HetR degradation due to PatS- and HetN-derived signals.

### The equations

We consider a chain of Ω cells, with Ω fixed. Denoted by *R*_*i*_, *S*_*i*_, and *N*_*i*_ are one individual of species HetR, PatS, and HetN, respectively. The index *i* runs from 1 to Ω and identifies the cell to which the individual belongs. The three species are produced at constant constitutive rates, here exemplified via the following chemical equations:

$$\begin{array}{l} \emptyset \overset{\alpha_{R}}{\to }R_{i}\\ \emptyset \overset{\alpha_{S}}{\to }S_{i}\\ \emptyset \overset{\alpha_{N}}{\to }N_{i} \end{array}$$ (1)

Furthermore, *R*_*i*_ regulates itself by positive feedback [9, 53]

$$\emptyset \overset{\beta_{R}\frac{{\left(\frac{r_{i}}{V}\right)}^{2}}{K^{2}+{\left(\frac{r_{i}}{V}\right)}^{2}}}{\to }R_{i}$$ (2)

where *β*_*R*_ is the strength of the positive autoregulation of HetR and *K*^2^ is the dissociation constant of HetR dimers. We have assumed that the active form of HetR is dimeric [54], even though a tetrameric form has been detected recently [55]. Here, *r*_*i*_ denotes the total number of HetR molecules in cell *i*, and *V* stands for the volume of each cell.

HetR activates production of PatS [54], with strength *β*_*S*_

$$\emptyset \overset{\beta_{S}\frac{{\left(\frac{r_{i}}{V}\right)}^{2}}{K^{2}+{\left(\frac{r_{i}}{V}\right)}^{2}}}{\to }S_{i}$$ (3)

Note that there is no evidence in the literature for activation of HetN production by HetR. Additionally *R*_*i*_, *S*_*i*_, and *N*_*i*_ undergo degradation at constant rates

$$\begin{array}{ll} R_{i} & \overset{k_{R}}{\to }\emptyset \\ S_{i} & \overset{k_{S}}{\to }\emptyset \\ N_{i} & \overset{k_{N}}{\to }\emptyset \end{array}$$ (4)

PatS and HetN form complexes with HetR dimers, effecting post-transcriptional HetR degradation [15]

$$\begin{array}{ll} R_{i}+R_{i}+S_{i} & \overset{\mu_{S}}{\to }\emptyset \\ R_{i}+R_{i}+N_{i} & \overset{\mu_{N}}{\to }\emptyset \end{array}$$ (5)

These terms are analogous in form to the stoichiometric down-regulation of mRNA targets by small RNAs in bacteria [56, 57]. Molecules *S*_*i*_ and *N*_*i*_ can diffuse along the chain, yielding

$$\begin{array}{ll} S_{i}\overset{D_{S}}{\to } & S_{j}\\ N_{i}\overset{D_{N}}{\to } & N_{j} \end{array}$$ (6)

Here, *j* points to the cells adjacent to cell *i*. To proceed in the analysis, we introduce the discrete quantities *r*_*i*_, *s*_*i*_, and *n*_*i*_ to identify the total number of HetR, PatS, and HetN in each cell *i* at time *t*. The state of the system is therefore completely specified by the vector (**r**,**s**,**n**) of dimension 3Ω, where **r** = (*r*_1_…*r*_Ω_), **s** = (*s*_1_…*s*_Ω_) and **n** = (*n*_1_…*n*_Ω_).

Introduce *P*(**r**,**s**,**n**,*t*) to label the probability for the system to be in state (**r**,**s**,**n**) at time *t*. Transitions from one state to another are dictated by the chemical equations listed above. Following standard notation, we assign *T*(**r′**,**s′**,**n′**|**r**,**s**,**n**) to characterize the transition rate from state (**r**,**s**,**n**) to state (**r′**,**s′**,**n′**), which is compatible with the former. A complete account of the transition rates in the model is provided (S1 Text). Under the Markov approximation, the dynamics of the system is governed by a master equation which can be cast in the generic form

$$\frac{d}{dt}P(r,s,n,t)=\sum_{\begin{array}{c} r^{\prime },s^{\prime },n^{\prime }\neq r,s,n \end{array}}[T(r,s,n|r^{\prime },s^{\prime },n^{\prime })P(r^{\prime },s^{\prime },n^{\prime },t)-T(r^{\prime },s^{\prime },n^{\prime }|r,s,n)P(r,s,n,t)]$$ (7)

The master equation provides an exact description of the stochastic dynamics. In the limit where the volume of each cell *V* goes to infinity, the system becomes deterministic: the coupled dynamics of the continuous concentrations is described by a set of ordinary differential equations. The effect of fluctuations, stemming from the discreteness of the system and here exemplified by a finite carrying capacity *V*, can be in turn accessed by numerical simulations of the chemical reaction model via the Gillespie algorithm [58]. This method produces realizations of the stochastic dynamics that are formally equivalent to those found from the master equation. Notice that volume *V* solely enters the definition of the reaction rates associated to Eqs (2) and (3). The other rates are independent of *V*. The finite size parameter *V* is, however, present in the rate equations that follow the aforementioned chemical reactions, as we will make explicit (S1 Text). Analytical progress is also possible by invoking the so-called van Kampen system-size expansion. This amounts to effectively expanding the master equation in powers of *V*^−1/2^: to leading order (*V* → ∞), one obtains the deterministic equations, while next-to-leading contributions give finite *V* corrections. These latter take the form of linear stochastic differential equations that can be straightforwardly analyzed, especially in the case when the deterministic system has approached a stable fixed point.

Notice that *V* stands for the volume of individual cells, not the actual copy number of the regulator molecules. In the literature, the van Kampen expansion is often implemented by assuming the number *N* of interacting entities as the relevant control parameter. This is particularly convenient when the inspected population stays constant over time. Conversely, when *N* gets modulated in time by the imposed dynamics, it is customary to adopt *V* as the reference extensive quantity, which encodes for the characteristic size of the system [59–61]. *V* and *N* are mutually related through the number density, a (dimensional) constant that is not made experimentally available for *Anabaena*. It is therefore not possible, at least at present, to establish a quantitative bridge between *N* and *V*. This latter quantity has to be regarded as a free control parameter, which can be tuned at will so as to control the strength of the imposed demographic noise.

The van Kampen expansion is based on substituting the ansatz

$$\begin{array}{ll} & \frac{r_{i}}{V}=\phi_{i}+\frac{\xi_{1,i}}{\sqrt{V}}\\ & \frac{s_{i}}{V}=\psi_{i}+\frac{\xi_{2,i}}{\sqrt{V}}\\ & \frac{n_{i}}{V}=\eta_{i}+\frac{\xi_{3,i}}{\sqrt{V}} \end{array}$$ (8)

into the master Eq (7). Here, *ϕ*_*i*_, *ψ*_*i*_, and *η*_*i*_ stand for the deterministic concentrations, associated with cell *i*, while *ξ*_1,*i*_,*ξ*_2,*i*_,*ξ*_3,*i*_ are the corresponding stochastic terms, triggered by finite size corrections. In the next section, we will focus on the deterministic mean field limit and investigate the conditions that underlie the process of pattern formation à la Turing. We will then turn to analyze the linear stochastic differential equations, obtained at the next-to-leading order of the van Kampen expansion, to report on the emergence of stochastic self-organized patterns. The derivation is lengthy (S1 Text).

## The deterministic limit

At the leading order in the van Kampen system-size expansion, we get the following set of 3Ω deterministic equations

$$\{\begin{array}{c} \begin{array}{ll} {\dot{\phi }}_{i} & =\alpha_{R}-k_{R}\phi_{i}+\beta_{R}\frac{\phi_{i}^{2}}{K^{2}+\phi_{i}^{2}}-2\mu_{S}\phi_{i}^{2}\psi_{i}-2\mu_{N}\phi_{i}^{2}\eta_{i}\\ {\dot{\psi }}_{i} & =\alpha_{S}-k_{S}\psi_{i}+\beta_{S}\frac{\phi_{i}^{2}}{K^{2}+\phi_{i}^{2}}-\mu_{S}\phi_{i}^{2}\psi_{i}+D_{S}\sum_{j}^{\Omega }\Delta_{ij}\psi_{j}\\ {\dot{\eta }}_{i} & =\alpha_{N}-k_{N}\eta_{i}-\mu_{N}\phi_{i}^{2}\eta_{i}+D_{N}\sum_{j}^{\Omega }\Delta_{ij}\eta_{j} \end{array} \end{array}$$ (9)

where Δ_*ij*_ = *W*_*ij*_ − *k*_*i*_*δ*_*ij*_ denote the entries of the discrete Laplacian operator Δ and *k*_*i*_ stands for the connectivity of node (cell) *i*. *W*_*ij*_ stand for the elements of the adjacency matrix (see S1 Text) associated with the one-dimensional chain that defines the spatial support of the model. The cells are organized in a one-dimensional lattice with nearest neighbors coupling. Each cell has two adjacent neighbors, except for those positioned at the edges. This latter boundary condition is embedded in the definition of matrix W. The deterministic model displayed above supports a large gallery of peculiar dynamical states, which range from multiple homogeneous attractors to non-homogeneous equilibria. Starting from these premises, we are here interested in studying diffusion-driven instabilities of the Turing class: when perturbed by a nonhomogeneous tiny disturbance, homogeneous fixed points can turn unstable following a symmetry-breaking instability. The spatially extended motifs that are self-consistently established because of the interplay between reaction and diffusion terms might explain the spontaneous differentiation from vegetative to heterocyst cells along the *Anabaena* filaments. We will begin by addressing the analysis in the idealized deterministic setting and then proceed towards the more realistic stochastic scenario. In the following, we will label with (ϕ*,ψ*,η*) the reference homogeneous equilibrium of Eq (9). Details on the procedure adopted to single out the sought equilibrium are provided in S1 Text.

### The Turing instability

To find out the conditions that underlie a deterministic Turing instability, we carry out a linear stability analysis around the homogeneous fixed point (ϕ*,ψ*,η*). We hence introduce small, nonhomogeneous perturbations (*δϕ*_*i*_,*δψ*_*i*_,*δη*_*i*_) and linearize around the fixed point, following [62] (see S1 Text). To solve the obtained linear system requires expanding the imposed perturbation on the complete basis formed by the eigenvectors of the Laplacian operator Δ. The procedure yields the following matrix:

$$Q=\left(\begin{array}{ccc} F & -\mu_{S}{\left(R^{*}\right)}^{2} & -\mu_{N}{\left(\phi^{*}\right)}^{2}\\ G & -k_{S}-\mu_{S}{\left(\phi^{*}\right)}^{2} & 0\\ -2\mu_{N}\phi^{*}\eta^{*} & 0 & -k_{N}-\mu_{N}{\left(\phi^{*}\right)}^{2} \end{array}\right)+\left(\begin{array}{ccc} 0 & 0 & 0\\ 0 & D_{S} & 0\\ 0 & 0 & D_{N} \end{array}\right)\Lambda^{\left(\alpha \right)}$$ (10)

where Λ^(*α*)^, *α* = 1,…Ω stand for the real eigenvalues of the negative semidefinite matrix Δ. Here, $F=-k_{R}+\beta_{R}\frac{2\phi^{*}K^{2}}{{\left(K^{2}+{\left(\phi^{*}\right)}^{2}\right)}^{2}}-2\mu_{S}\phi^{*}\psi^{*}-2\mu_{N}\phi^{*}\eta^{*}$ and $G=\beta_{S}\frac{2\phi^{*}K^{2}}{{\left(K^{2}+{\left(\phi^{*}\right)}^{2}\right)}^{2}}-2\mu_{S}\phi^{*}\psi^{*}$. The eigenvalue of *Q* with the largest real part, *λ*_*max*_(Λ^(*α*)^), defines the dispersion relation and ultimately determines the response of the system to the imposed external perturbation. If the dispersion relation is positive over a finite domain in Λ^(*α*)^, the perturbation gets exponentially enhanced and eventually materializes in asymptotic patterns, whose spatial characteristics are set by the excited discrete wavelengths Λ^(*α*)^. When *λ*_*max*_ < 0, the perturbation fades away and the system converges to the unperturbed homogeneous solution.

**Fig 3:**
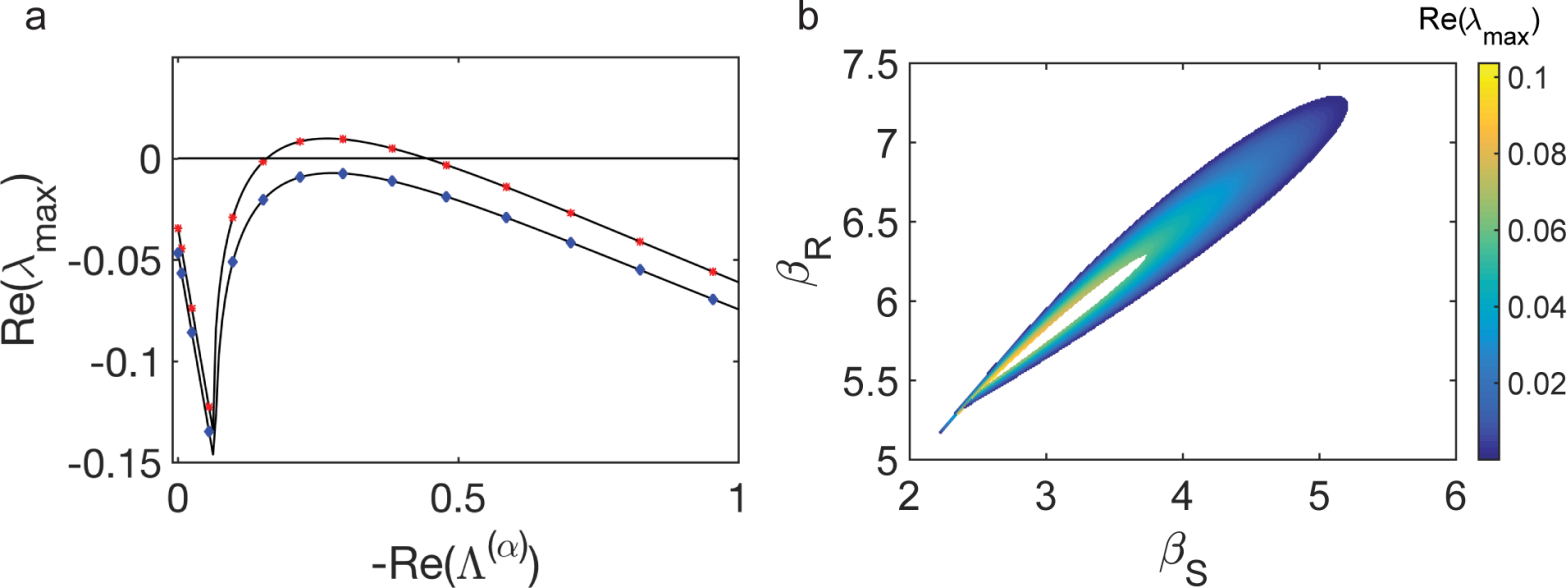
Conditions for a deterministic Turing instability. (**a**) Dispersion relations for *β*_*R*_ = 6.5 and *β*_*S*_ = 3.65 (blue diamonds) and *β*_*R*_ = 6.5 and *β*_*S*_ = 3.7 (red stars). The data used in this figure are included in S1 Data. (**b**) Region in the plane (*β*_*S*_,*β*_*R*_) where the maximum of *λ*_*Re*_(Λ^(*α*)^) is positive and the equilibrium point is stable, for a ratio of diffusion coefficients $\frac{D_{S}}{D_{N}}=3$. Parameters are set as *k*_*R*_ = 0.2, *α*_*R*_ = 0.2, *K* = 2, *k*_*S*_ = 0.1, *α*_*S*_ = 0.1, *μ*_*S*_ = 0.1, *k*_*N*_ = 0.7, *α*_*N*_ = 0.3, *μ*_*N*_ = 3, *D*_*S*_ = 3, *D*_*N*_ = 1, and Ω = 40.

At variance with the conventional Turing analysis, the patterns are here established on a discrete and finite support. The dispersion relation that applies to the limiting continuum setting can be readily recovered by replacing Λ^(*α*)^ with −*k*^2^, *k* being the usual spatial Fourier frequency. The discrete dispersion relation *λ*_*max*_(Λ^(*α*)^) results in a collection of Ω points distributed on the smooth profile that is obtained under the idealized continuum representation. In Fig 3A, the dispersion relations are plotted for two distinct values of *β*_*S*_. Symbols refer to the discrete dispersion relation, while the solid lines stand for their corresponding continuum analogues. To access analytically the conditions for the onset of the instability, one can operate under the continuum approximation and adapt the prototypical Turing calculation to the case of interest where three species are made to mutually interact (see S1 Text). We now freeze all parameters to nominal values except for *β*_*S*_ and *β*_*R*_. The colored region of Fig 3B denotes the portion of the plane (*β*_*S*_,*β*_*R*_) where the instability can take place. In this region, the maximum of the dispersion relation is positive. We stress that with the exception of experimental measurements of the in vitro affinity of the RGSGR peptide to HetR [63], there are no published experimental data to guide the choice of parameter values in our model. The smaller the ratio *D*_*S*_/*D*_*N*_ is, the narrower is the instability region (see S2 Fig when the ratio is equal to one).

**Fig 4:**
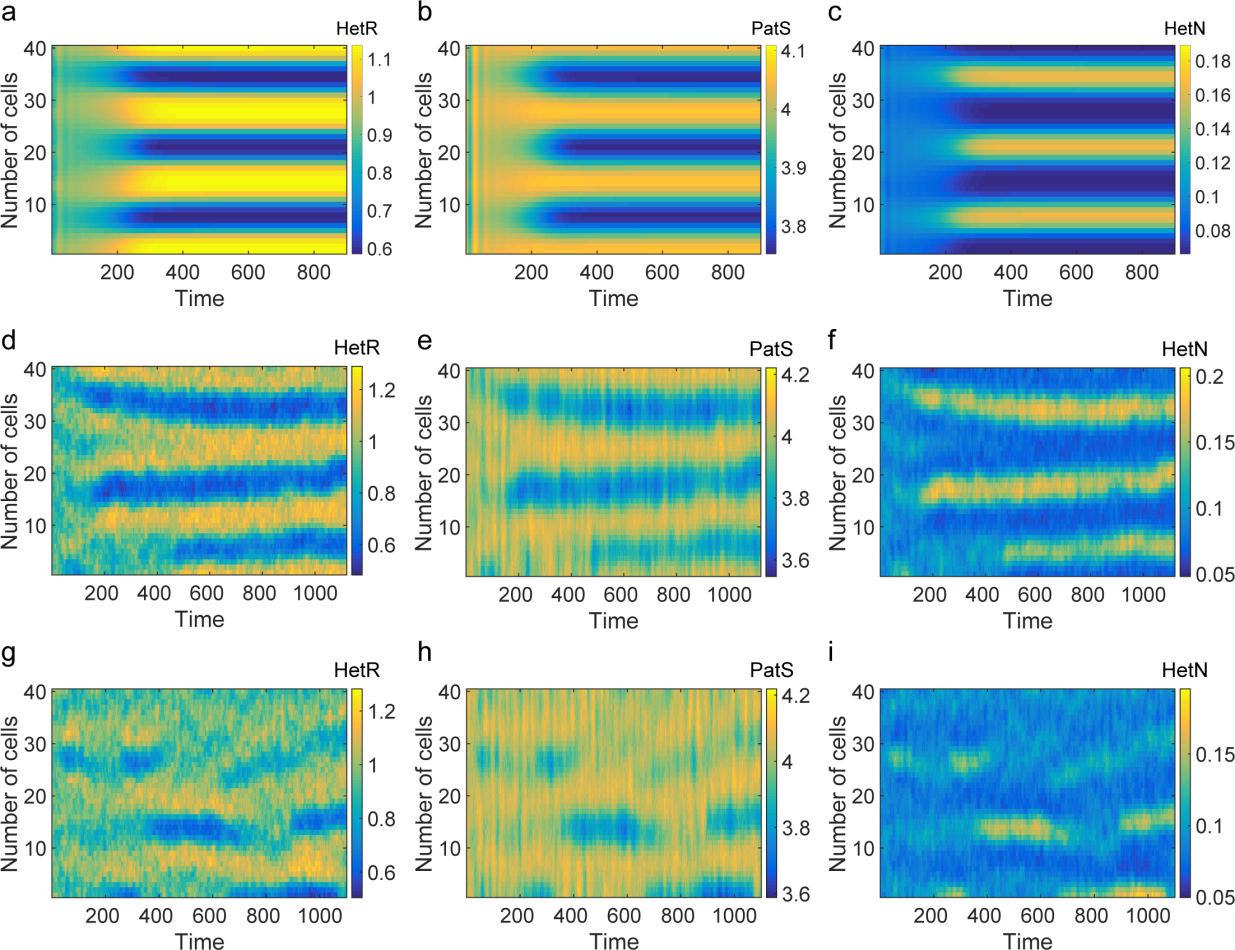
Turing patterns inside and outside the instability region. (**a**–**c**) Numerical integration of Eq (9). (**d**–**f**) Stochastic simulations using the Gillespie algorithm. Parameters correspond to those used to compute the red stars curve of Fig 3A and apply to all panels from (a) to (f). (**g**–**i**) Stochastic Turing patterns corresponding to the blue diamonds curve of Fig 3A. For all panels, Ω = 40 and *V* = 5000.

To test the validity of the theoretical predictions, we have numerically integrated Eq (9). Initializing the concentration of the species at the stable homogeneous configuration after the injection of small perturbations, the system self-organizes and displays Turing patterns (Fig 4A–4C). The patterns exhibit distinctive characteristic features that reflect the specific interactions at play between the different microscopic actors. A region with a high density of HetR induces an analogous crest in the concentration of PatS. This represents the nonlinear, sigmoidal activation of PatS by HetR. Conversely, high concentrations in HetR induces a depletion of HetN content, due to the nonlinear decay term in the equation for HetN (third equation in Eq (9)). The typical separation between adjacent peaks can range from a few to tens of cells depending on the selected parameters and includes as a possible setting the experimentally observed scenario. A notable feature of the deterministic patterns we obtain is that the modulation in concentration of the different regulators is smooth and varies over a number of cells, whereas in developmental patterns in *Anabaena*, variation is more abrupt and localized on single cells (see, e.g., Fig 2). Furthermore, the model does not reflect the temporal differences in the onset of production of PatS and HetN. These behaviors stem from the fact that we have not enforced any distinction between vegetative and heterocyst cells. Commitment and the ensuing differentiation into a heterocyst state must be governed by downstream genetic factors not included in our model, such as HetP and other proteins that share a functional domain with it, as discussed recently [12]. We posit that these genetic factors could also effectively stabilize transient noisy patterns outside the region in parameter space where the deterministic Turing instability takes place. In the following, we will show that stochastic patterns can indeed develop when the dispersion relation would predict the homogeneous fixed point to be stable. Endogenous demographic noise seeds a spatial modulation in the concentration of regulators, with nominal density peaks and associated characteristic spacing that quantitatively resemble those obtained when operating inside the region of deterministic order. The intrinsic ability of the stochastic system to self-organize beyond the boundary of the classical Turing instability, followed by fixation of transient patterns as a result of a commitment process that is triggered by sufficiently large concentration gradients in the biological system, can result in an extraordinarily robust mechanism for pattern formation in *Anabaena*.

## Stochastic Turing patterns

The role played by demographic noise can be appreciated by performing numerical simulations at finite *V* using the Gillespie's algorithm [58]. Fig 4D–4F displays stochastic patterns, obtained for the same choice of parameters as in the deterministic setting (*V* → ∞) (see Fig 4A–4C). The corresponding dispersion relation has a positive real part (upper curve in Fig 3A), and the recorded patterns display many similarities with their deterministic analogues. We now turn to stochastic simulations for a choice of parameters for which the deterministic Turing instability cannot develop (lower curve in Fig 3A). The resulting patterns (Fig 4G–4I) are less defined but still visible to the eye, even when the system is initialized outside the region of deterministic order. The stochastic forcing produces sustained oscillations in space, which give rise to spatial patterns with distinct characteristic features, reminiscent of those obtained inside the region of deterministic order. To quantitatively substantiate this claim, we consider the next-to-leading approximation in the van Kampen expansion to obtain a closed analytical characterization of the stochastic fluctuations. Collecting terms in the expansion that scale proportionally to 1/*V*, one finds a Fokker–Planck equation for the distribution of fluctuations (see S1 Text for details about the derivation). The Fokker–Plank equation is equivalent to the following system of coupled linear Langevin equations:

$$\frac{d\xi_{q,i}}{d\tau }=\sum_{l=1}^{3}\sum_{j=i}^{\Omega }M_{ql,ij}\xi_{l,j}+\chi_{q,i}$$ (11)

where *ξ*_*q*,*i*_ denotes the fluctuations that affect the concentration of species *q* on site *i*. Here, *q* = 1 (HetR), 2 (PatS), 3 (HetN), and *χ*_*q*,*i*_ is a Gaussian white noise, with zero mean and correlator ⟨*χ*_*q*,*i*_(*τ*)*χ*_*l*,*j*_(*τ*')⟩ = *B*_*ql*,*ij*_*δ*(*τ*−*τ*'). The 3Ω×3Ω matrices *M* and *B* are given in S1 Text. They depend on the solution of the deterministic equation and so are in principle time dependent. However, we are here interested in fluctuations about the stationary state: The mean field concentrations can be set to their constant equilibrium values (ϕ*,ψ*,η*), and consequently, the matrices *M* and *B* lose their time dependence. They also have a nontrivial spatial dependence through the presence of the discrete Laplacian operator. To proceed in the calculation, we introduce an appropriate transform inspired by standard Fourier analysis but specifically designed to account for the discrete nature of the spatial support, including the enforced boundary conditions [64]. Label with **v**^(*α*)^ the eigenvector of the discrete Laplacian operator Δ, relative to the eigenvalue Λ^(*α*)^. The temporal and spatially discrete transform ${\tilde{f}}_{\alpha }(\omega )$ of a generic function *f*_*i*_(*t*) is defined as ${\tilde{f}}_{\alpha }(\omega )=\int_{0}^{+\infty }d\tau \sum_{j=1}^{\Omega }f_{j}(\tau )v_{j}^{\left(\alpha \right)}e^{i\omega \tau }$. When applied to the Eq (11), the transform disentangles the spatial coupling and yields the closed solution

$${\tilde{\xi }}_{q,\alpha }=\sum_{l=1}^{3}F_{ql}^{-1}{\tilde{\chi }}_{l,\alpha }$$ (12)

where **F** is $\left(-i\omega I-M^{\left(NS\right)}-M^{\left(SP\right)}\Lambda^{\left(\alpha \right)}\right)$ and *M*^(*NS*)^ (*M*^(*SP*)^, respectively) stand for the nonspatial (spatial, respectively) component of *M*, as defined in S1 Text. We can then compute the power spectrum of fluctuations as

$$P_{q}(\omega ,\Lambda^{\left(\alpha \right)})=\langle |{\tilde{\xi }}_{q,\alpha }(\omega {)|}^{2}\rangle =\sum_{l,m=1}^{3}F_{ql}^{-1}(B_{ml}^{\left(NS\right)}+B_{ml}^{\left(SP\right)}\Lambda^{\left(\alpha \right)})(F^{†}{)}_{lq}^{-1}$$ (13)

where (**F**^†^)^−1^ denotes the inverse of the adjoint of **F**. The non-spatial (*B*^(*NS*)^) and spatial (*B*^(*SP*)^) contributions to matrix *B* appear explicitly (see S1 Text).

**Fig 5:**
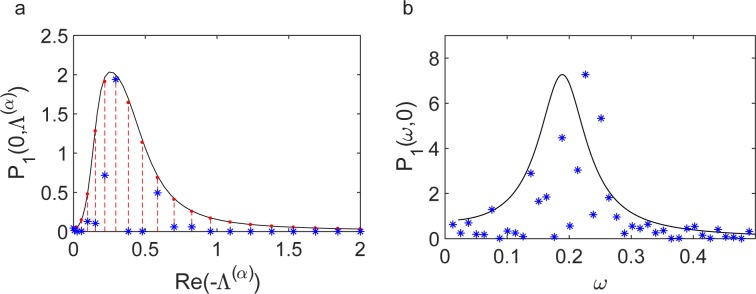
Power spectrum of the fluctuations. (**a**) Power spectrum evaluated at *ω* = 0 as a function of −*Re*(Λ^(*α*)^). The solid black line corresponds to the theoretical prediction on a continuous domain, and red points correspond to the theoretical formula using a filament with 40 cells. Blue stars stand for the power spectrum obtained from just one realization of the Gillespie algorithm. In the stochastic simulation, we used 40 cells and analyzed the recorded signal in the time interval *τ* [400,900] (see Fig 4G–4I). (**b**) Power spectrum evaluated at *Re*(Λ^(*α*)^) = 0 as a function of *ω*. The solid black line is the analytical theory, while blue stars correspond to the stochastic simulation. Theoretical and numerical profiles are normalized so as to yield the same maximum value (using the discrete theoretical curve for the spatial slice). Parameters are the same as those in Fig 4G–4I. The data used in this figure are included in S1 Data.

In Fig 5A, we plot *P*_1_(0,Λ^(*α*)^) as a function of the discrete wavelength Λ^(*α*)^, for the same parameter values as in the simulations of Fig 4G–4I. A clear peak is displayed, implying that noise promotes the spontaneous selection of a leading wavelength in the emerging patterns. More importantly, the latter coincides with the wavelength that becomes unstable when the system is taken inside the corresponding region of the deterministic instability. In this latter case, the pattern characteristics are shaped by the wavelength that maximizes the unstable dispersion relation (upper curve in Fig 3A). In Fig 5B, we plot *P*_1_(0,*ω*) versus the time frequency *ω*.

To test the correctness of the theory, we carried out stochastic simulations using the Gillespie algorithm. The numerical power spectrum is reconstructed from an individual realization by applying the generalized transform introduced above. In Fig 5A and 5B red stars refer to the power spectra obtained from just one realization of the stochastic dynamics. The peaks are located as predicted by the theory, implying that the stochastic-driven patterns formation mechanism works efficiently for single *Anabaena* filaments. By averaging over many independent realizations, one recovers a perfect match with theoretical curves. In other words, for our model of *Anabaena*, the number of excited modes is sufficiently small that the analysis of the power spectrum proves successful in predicting the asymptotic outcome, as observed in the interesting paper by Maini and colleagues [65].

**Fig 6:**
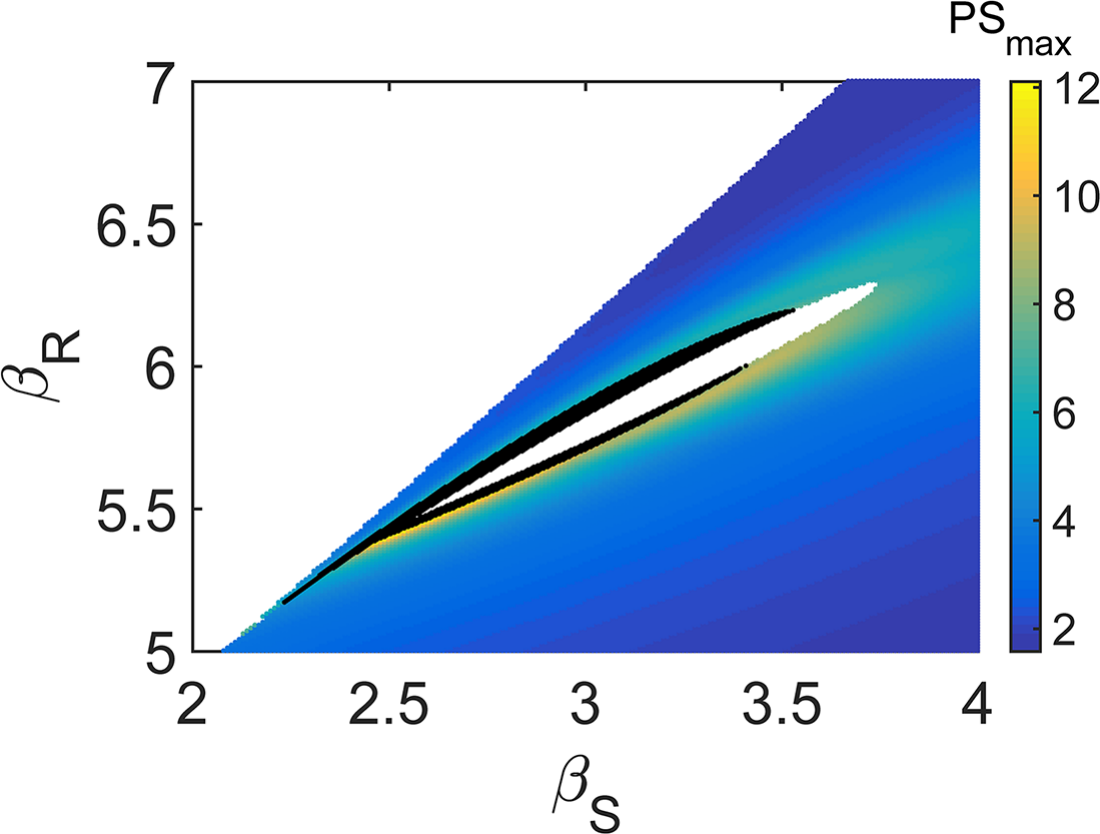
Region of stochastic versus deterministic Turing instability. Region in the plane (*β*_*S*_,*β*_*R*_) where the power spectrum of HetR fluctuations displays a localized maximum. The color code reflects the height of the peak. The domain filled in black denotes the deterministic instability region (see S2B Fig) Parameters are set as *k*_*R*_ = 0.2, *α*_*R*_ = 0.2, *K* = 2, *k*_*S*_ = 0.1, *α*_*S*_ = 0.1, *μ*_*S*_ = 0.1, *k*_*N*_ = 0.7, *α*_*N*_ = 0.3, *μ*_*N*_ = 3, *D*_*S*_ = 4, *D*_*N*_ = 4, and Ω = 40.

Summing up, stochastic corrections stemming from the finite size can eventually produce macroscopically ordered structures that quantitatively resemble those obtained under the deterministic Turing scenario. The differentiation into a heterocyst cell is then effected by downstream genetic factors, which get locally activated in response to large gradients of concentrations that result from the noisy component of the dynamics. According to the noise-driven mechanism proposed here, patterns can also arise when the diffusion constants are comparable *D*_*S*_ ∼ *D*_*N*_, as demonstrated in S3 Fig. Indeed, the region of noise-driven instability is considerably larger than the corresponding deterministic region, as clearly depicted in Fig 6.

## Growing domain

**Fig 7:**
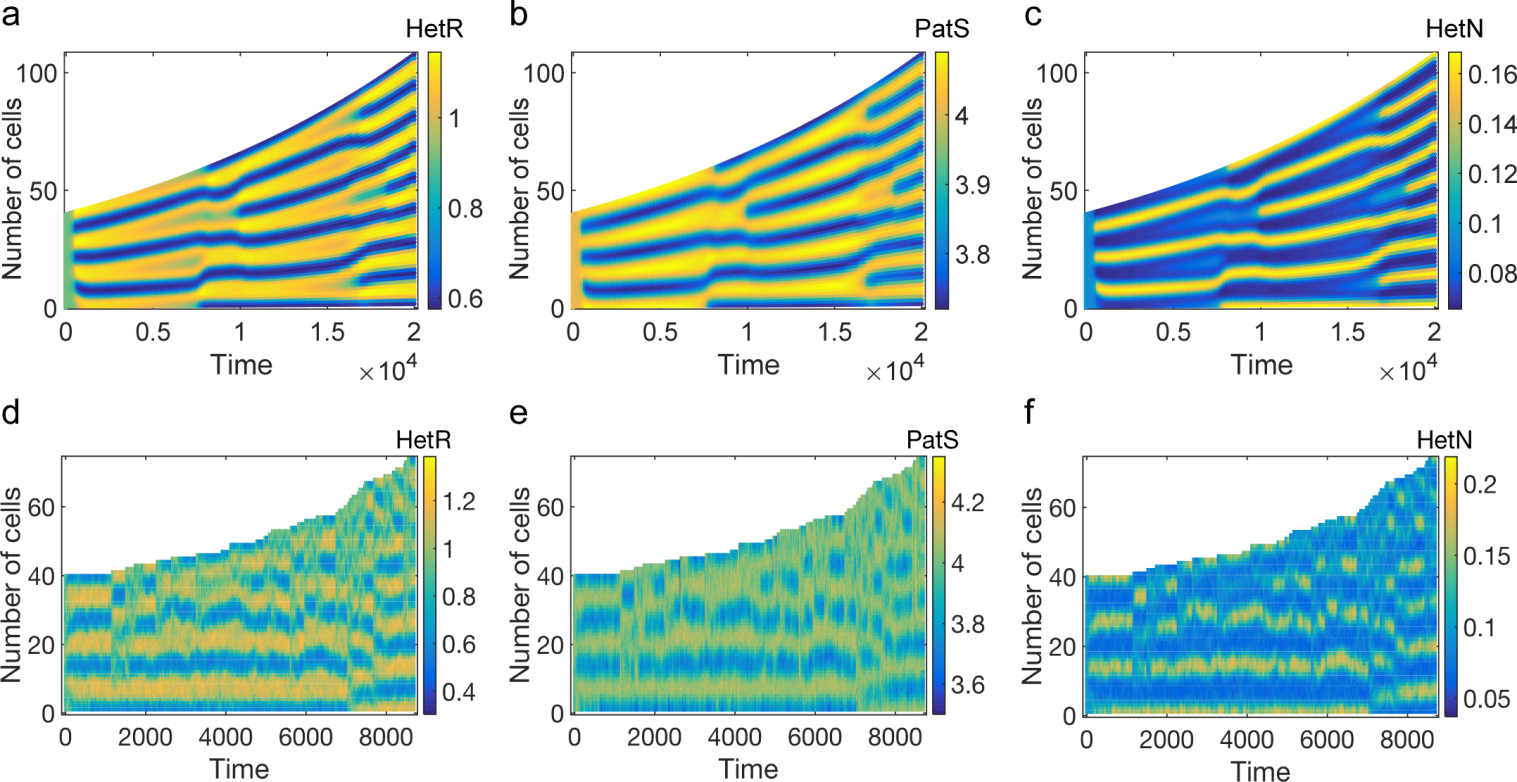
Patterns on a growing filament. (**a–c**) Deterministic patterns obtained by integrating Eq (39) in section 4 of S1 Text. (**d–f**) Gillespie simulations of a growing filament taking demographic noise into account. Parameters are set as those of Fig 4A and 4B, while the growth rate *ρ* is set to 10^−8^.

The stochastic model that we have introduced can be modified to account for the growth of filaments, due to cell duplication [66, 67]. The number of cells, Ω, is hence a stochastic variable, and the master equation should be modified to reflect this additional ingredient. More specifically, label with *P*(**r**,**s**,**n**,Ω,*t*) the probability of seeing the system in a configuration specified by the state vectors (**r**,**s**,**n**), with Ω cells, at time *t*. Consistent with the above, we do not enforce on the model the differentiation from vegetative to heterocysts cells. We are in fact solely interested in the spontaneous emergence of patterned motifs in the regulators’ concentration that might anticipate the subsequent, genetic-driven commitment. For this reason, all cells composing the filaments can experience the duplication event. To implement the growth mechanism, the state vector **r** (**s** and **n**, respectively) is assumed to be of arbitrary size: the first Ω entries are different from zero and reflect the actual concentration in HetR (PatS and HetN, respectively). All components *r*_*i*_ (*s*_*i*_ and *n*_*i*_, respectively) with *i* > Ω are identically equal to zero and get progressively populated as the filament grows. Under these assumptions, the master equation that rules the dynamics of the system on a growing support is obtained by adding to the right hand side of Eq (7) the terms

$$\sum_{\begin{array}{c} r^{\prime },s^{\prime },n^{\prime }\neq r,s,n \end{array}}\left[T_{dupl}(\Omega |\Omega -1)P(r^{\prime },s^{\prime },n^{\prime },\Omega ,t)-T_{dupl}(\Omega +1|\Omega )P(r,s,n,\Omega ,t)\right]$$ (14)

constrained to act on the first Ω cells of the lattice. Here, *T*_*dupl*_(⋅|⋅) stands for the duplication rate that we set to a constant, *ρ*. For the sake of simplicity, in the expression for *T*_*dupl*_, we do keep explicit track of the number of regulators. In our analysis, we assume that the amounts of HetN, PatS, and HetR get equally shared between daughter cells. The same qualitative conclusion as reported above holds, however, if binomial splitting of the genetic material is instead considered. From the master equation, one can readily obtain the 3Ω ordinary differential equations that govern the evolution of the concentration in the deterministic limit (see S1 Text) where Ω = Ω_0_*e*^*ρVτ*^, Ω_0_ labeling the number of cells that initially compose the *Anabaena* filament. In the limit of a continuous spatial support, the growth yields an additional linear decay term, which scales with *ρ*, and time-modulated diffusion constants (see S1 Text). Deterministic and stochastic simulations are reported in Fig 7A–7C and 6D–6F. Patterns are established and subsequently maintained with unaltered spacing, by successive insertion of high-density regions, where heterocysts would presumably localize. When the separation between heterocysts cells becomes large enough, the system self-organizes so as to enhance the concentration of HetR near the middle of the interval. This could anticipate the insertion of a new intercalary heterocyst to preserve the characteristic spacing, as seen in the experiments. The number of linearly unstable spatial modes increases with the filament size (see S4 Fig), a mechanism that possibly facilitates the maintenance of the patterns. In the simulations reported in Fig 7, we assumed that deterministic patterns could develop on the initial filament of size Ω_0_. The conclusions remain unchanged, however, if patterns initially assumed fixed domain are instead stochastic in nature.

## Discussion

We have studied a theoretical model that describes the nearly periodic patterns observed when *Anabaena* filaments are subject to nitrogen deprivation. Our experiments, as well as those of others, informed our choice of dynamical variables and constrained the mathematical form their mutual interaction takes. Our goal has been to explore the consequences of our choice in an attempt to discover conditions and mechanisms for pattern formation and maintenance, rather than to fit specific model parameters and match experimental measurables such as heterocyst spacing distributions. To keep the model analytically tractable, only three dynamical variables have been considered. Thus, we have refrained from including effects such as the immunity of HetR against inhibition by PatS produced in the same cell as mediated by the HetC and PatA proteins [17, 18, 68]; biased inheritance of factors such as PatN that may influence a cell’s decision to differentiate [69]; enforcing a distinction between vegetative and heterocyst cells [44]; and factors such as HetP that modulate commitment to differentiation into a heterocyst state [12].

In an endeavor to choose proper dynamical variables that capture essential features of development in *Anabaena*, we have revisited the question of the need of two inhibitors of the master regulator of differentiation HetR. Our experiments challenge a clear-cut separation of the involvement of PatS and HetN in de novo pattern formation and pattern maintenance (often found in the literature), respectively, and suggest instead that both PatS and HetN are present during pattern maintenance but have different spatiotemporal roles, as also proposed recently [48]. These experiments clearly establish that significant HetN production takes place only after a cell has committed to a heterocyst fate, recapitulating previous results [21]. HetN production sets up an inhibitory signal gradient that decays with distance from heterocysts and that prevents the formation of new intercalary heterocysts close to existing ones. Remarkably, activation of HetN production takes place with high temporal precision after the onset of the decline in photosynthetic autofluorescence and displays switch-like characteristics. Of note, the involvement of HetP and its homolog regulators during commitment has also been shown to result in a switch-like output [12]. In contrast, lateral inhibition processes observed in clusters of cells near the middle of vegetative intervals (where levels of both fixed nitrogen products and the HetN-derived inhibitory signal are low) take place before the observed decline in autofluorescence. We posit that only PatS can mediate this lateral inhibition process, as HetN is only produced at significant levels after the reduction in autofluorescence. This difference in timing of activation has been noted earlier [70]. Thus, PatS functions similarly both during de novo pattern formation, when primordial fluctuations in gene expression that exist under nitrogen-replete conditions are amplified to create an initial pattern, as well as during pattern maintenance, in the initial stages of incipient intercalary heterocyst formation [71]. It is possible that the transient activation of PatS required to resolve small clusters of cells exhibiting *hetR* activation into a single heterocyst may have prevented its detection during pattern maintenance in earlier works [72], and to claims of a diminished role for PatS during the pattern maintenance stage [15, 73]. The different spatiotemporal roles of PatS and HetN in the picture proposed here are reminiscent of the ideas embodied in the models of Turing and Wolpert [22, 74] and illustrate that these ideas are not mutually exclusive and can work together [75]. Together, the above considerations led us to choose HetR, PatS, and HetN as dynamical variables, in accordance with a previous numerical study of a reaction–diffusion model in which roles of PatS and HetN similar to the ones envisioned here were proposed [48]. Being minimal, any perturbation to the model, such as removing one of the inhibitors, the positive autoregulation of HetR, or cell–cell communication, will destroy any Turing instability. This precludes a comparison with deletion mutants in *Anabaena* [11].

In contrast to previous models of *Anabaena* development, we have taken demographic noise into account explicitly by starting from master equations that embody production and degradation of individual molecules. This approach is fully justified by our measurements of a small copy number of GFP molecules produced from the *hetR* promoter, together with the significant levels of cell–cell heterogeneity (35%) measured previously for the same promoter, under nitrogen-rich conditions [11]. A linear stability analysis of the steady-state solutions of the set of reaction–diffusion equations obtained in the deterministic limit of a linear noise approximation to the master equations leads to a region of instability in which deterministic Turing patterns form, provided *D*_*S*_ > *D*_*N*_, in accordance with a previous numerical study [48]. However, this region shrinks severely as *D*_*S*_ → *D*_*N*_, a limit that may be relevant to the biology of *Anabaena*. Much is known about the PatS inhibitory signal. The pentapeptide RGSGR in the C-terminal 5 amino acids of PatS prevents the DNA-binding activity of HetR in vitro [54]; its addition to the medium prevents differentiation of heterocysts [72] and therefore has been thought to constitute the PatS-derived signal. More recently, the accumulation of an RGSGR-containing product in cells adjacent to proheterocysts was detected by immunofluorescence, with a gradient extending over 5–6 cells [13, 14]. The findings also indicated that an octapeptide containing the RGSGR motif was active as an inhibitor. In contrast to the above, less is known about the molecular identity of the HetN inhibitory signal. HetN bears an internal ERGSGR sequence that is identical to the C-terminal sequence of PatS, and deletion of this sequence results in the appearance of multiple contiguous heterocysts in the second round of heterocyst formation [20, 21]. The existing evidence argues in favor of the HetN-derived signal being a peptide resulting from processing of the full protein that consists of little more than the RGSGR motif and which is transferred between cells via SepJ and/or FraC/FraD septal proteins [20, 21]. Taken together, the above considerations and parsimony lead us to the notion that the diffusivities of PatS- and HetN-derived signals are comparable and thus that the limit *D*_*S*_ ≈ *D*_*N*_ likely reflects the biology of *Anabaena*. A direct consequence of this notion is that within the framework of the deterministic limit of our model, Turing patterns are unlikely to arise.

Remarkably, and in line with previous theoretical investigations of model systems [32–34], the next-to-leading order in the linear noise approximation clearly demonstrates the formation of stochastic Turing patterns outside the region in parameter space where deterministic patterns form. Gillespie simulations show that these patterns are clearly visible, in spite of their inherently transient character. Furthermore, they do arise when *D*_*S*_ is close to *D*_*N*_, suggesting a novel robust scenario for the formation and maintenance of developmental patterns in *Anabaena*. These may be the result of transient stochastic patterns that arise from the resonant amplification of demographic noise outside the region of deterministic instability. When fluctuations in HetR concentration in particular cells reach sufficiently high levels, irreversible commitment to the formation of heterocysts may be triggered by downstream genetic processes, thereby stabilizing the transient stochastic patterns. In support of this notion, it has been observed experimentally that overexpression of HetR suffices to trigger differentiation and the formation of heterocysts, even under repressing, nitrogen-replete conditions [10]. A division of development into patterning followed by commitment stages has also been considered recently [12]. Furthermore, our measurements of low copy numbers of GFP molecules produced by the *hetR* promoter and the significant levels of expression noise measured under nitrogen-replete conditions [11] confirm that demographic stochasticity is significant in this system and justifies our approach. Overall, this scenario is considerably more robust than the classical Turing mechanism, as stochastic patterns form in a much larger region of parameter space than their deterministic counterparts and do not require large differences in diffusivities. Including time delay prior the initiation of HetN production constitutes a possible avenue of further investigation. Time delay is in fact known to impact the process of pattern formation. In [65], typical Turing models are reported to lose robustness with the inclusion of delays. On other occasions, however, time delay can facilitate the onset of the instability [76, 77]. Gauging the impact of the delay for the problem at hand is left for future analysis. The robust scenario we propose is not specific to *Anabaena*. We can speculate that such a mechanism can be active in situations in which a pattern arises from the amplification of gene expression fluctuations and not when a morphogen gradient is imposed, e.g., in a *Drosophila* embryo.

We stress that a restricted model that includes only nondiffusing HetR and diffusing PatS cannot yield nonhomogeneous patterns, even when demographic noise as well as domain growth are included. However, small, nonzero levels of *hetN* expression under nitrogen-replete conditions have been reported recently [21, 78], suggesting that the three-variable model that includes *R*, *S*, and *N* may be applicable to de novo pattern formation as well. In this context, we note that in addition to *patS* and *hetN* genes, two other genes have been found to encode the RGSGR motif [73].

An interesting feature emerging from the analysis of the effects of filament growth in the deterministic limit of our model is that the number of linearly unstable spatial modes increases with filament length (see S1 Text). This finding is in line with the analysis of model systems [67]. Thus, growth promotes the instability of a homogeneous state and the formation of spatial patterns [79]. However, a linear stability analysis proved hard to carry out because differentiation and growth take place with similar temporal timescales, and incorporating growth as a function of time remains a challenging task for the future.

To sum up, our work highlights the essential role demographic noise plays in both the robust formation and maintenance of developmental patterns in *Anabaena*. Far from being a passive byproduct of molecular processes, fluctuations in copy numbers are used actively by this organism of primitive origin in order to seed and maintain developmental patterns and thus solve the incompatibility between nitrogen fixation and photosynthesis. Our model constitutes the first example of the applicability of stochastic Turing patterning in the context of morphogenesis and, together with examples from ecology and epidemics [80], underscores the generality of this robust mechanism in biological pattern formation.

## Methods

### Strains

The strains used in this study were obtained from conjugation with the wild-type *Anabaena* sp. (also known as *Nostoc* sp.) strain PCC 7120 as recipient; strain CSL64 bearing a chromosomally encoded *PhetR-gfp* transcriptional fusion; and strain CSL108 bearing a translational *hetN-gfp-mut2* fusion as the only HetN version in a wild-type background. Both strains have been reported previously [11, 21]. *Anabaena* producing GFP from a *PpatS-gfp* transcriptional fusion in a strain PCC 7120, *α* megaplasmid has been constructed and reported previously (CSVM17) [13, 81]. We note that all fusions preserve the native ribosome binding site of each gene, and therefore, they faithfully report on the physiological time of expression of each gene.

### Experiments

Strains were grown as described previously [82]. When required, antibiotics, streptomycin sulfate (Sm), and spectinomycin dihydrochloride pentahydrate (Sp) were added to the media at final concentrations of 2 *μg*/*mL* for liquid and 5 *μg*/*mL* for solid media. The densities of the cultures were adjusted so as to have a chlorophyll *a* content of 2–4 *μg*/*mL* 24 h prior to the experiment following published procedures [83]. For time lapse measurements, filaments grown in BG11_0_ + ammonium medium (in the presence of Sm and Sp for the CSL mutants) containing 2–3 *μg*/*mL* of chlorophyll a were harvested, washed three times with nitrogen-free (BG11_0_) medium, and concentrated 50 fold. An agarose low-melting gel pad (1.5%) in BG11_0_ medium with 10 mM NaHCO_3_ was made on a glass microscope slide. About 5 *μL* of culture were pipetted onto the pad and covered with a #0 mm coverslip, and this device was then placed on the microscope. *Anabaena* filaments within a device were followed as they developed, at 30°C in light. Filament growth and development within devices are similar to those in bulk cultures (see section 1 of S1 Text). Images were taken every 30 min on a Nikon Eclipse Ti-E microscope controlled by the NIS-Elements software using a 60 N.A 1.40 oil immersion phase contrast objective lens (Nikon plan-apochromat 60 1.40) and an Andor iXon X3 EMCCD camera. All the filters used are from Chroma. The filters used were ET480/40X for excitation, T510 as dichroic mirror, ET535/50M for emission (GFP set), ET430/24x for excitation, 505dcxt as dichroic mirror, and HQ600lp for emission (chlorophyll set). Samples were excited with a pE-2 fluorescence LED illumination system (CoolLED).

To calibrate fluorescence levels in terms of absolute numbers of protein molecules, we followed published methods [52]. In short, the fluorescence level of the i-th cell *y*_*i*_ is proportional to the number of fluorescent molecules *n*_*i*_: *y*_*i*_ = *νn*_*i*_. The proportionality constant *ν* is given by an average involving the fluorescence levels of mother cells *f*_*i*_ and their respective daughters *f*_2*i*_ and *f*_2*i*+1_:

$$\nu =\left\langle {\frac{\left(f_{2i}-f_{2i+1}\right)}{f_{i}}}^{2}\right\rangle$$

Prior to the calculation of *ν*, a constant background stemming from the contribution of the autofluorescence of photosynthetic pigments in the same region of the spectrum was subtracted from the total GFP fluorescence signal in each cell. The background correction was measured in a wild-type *Anabaena* sp. PCC 7120 strain bearing no fluorescent reporter. The number of mother–daughter triplets used for the calculation was 51. The sum of the fluorescence levels of the daughter cells was smaller than that of the respective mother cell by about 2%, primarily due to photo-bleaching.

### Image segmentation

All image processing and data analysis was carried out using MATLAB (MathWorks). Filament and individual cell recognition was performed on phase contrast images using an algorithm developed in our laboratory. The program's segmentation was checked in all experiments and corrected manually for errors in recognition. The total fluorescence from GFP and chlorophyll channels of each cell, as well as the cell area, were obtained as output for further statistical analysis.

## Supporting information

S1 Fig Distribution of vegetative cell interval sizes between heterocysts in an *Anabaena* strain of wild-type background bearing a *PhetR-gfp* fusion. Filaments from ammonium-supplemented cultures were washed three times with BG11_0_ medium, resuspended in BG11_0_ medium, and grown in one of our devices prepared with BG11_0_ medium (see Methods). Filaments were followed in real time to ascertain which cells became heterocysts. Intervals (*n* = 248) were counted 24 h after nitrogen deprivation. The mean interval size is 9.3±0.5 cells, and error bars represent standard errors from five independent experimental runs. The data used in this figure are included in S1 Data.
(PDF)

S2 FigConditions for a deterministic Turing instability.(**a**) Dispersion relations for *β*_*R*_ = 5.69 and *β*_*S*_ = 2.99 (blue diamonds) and *β*_*R*_ = 5.82 and *β*_*S*_ = 2.99 (red stars). The data used in this figure are included in S1 Data. (**b**) Region in the plane (*β*_*S*_,*β*_*R*_) where the maximum of *λ*_*Re*_(Λ^(*α*)^) is positive, and the equilibrium point is stable for a ratio of diffusion coefficients $\frac{D_{S}}{D_{N}}=1$. Parameters are set as *k*_*R*_ = 0.2, *α*_*R*_ = 0.2, *K* = 2, *k*_*S*_ = 0.1, *α*_*S*_ = 0.1, *μ*_*S*_ = 0.1, *k*_*N*_ = 0.7, *α*_*N*_ = 0.3, *μ*_*N*_ = 3, *D*_*S*_ = 4, *D*_*N*_ = 4, and Ω = 40.(PDF)

S3 Fig Turing patterns inside and outside the instability region. (**a**—**c**) Numerical integration of Eq (9) of the main text. (**d**–**f**) Stochastic simulations using the Gillespie algorithm. Parameters correspond to those used to compute the red stars curve of Fig 3A and apply to all panels from (a) to (f). (**g**–**i**) Stochastic Turing patterns corresponding to the blue diamonds curve of Fig 3A. For all panels, Ω = 40 and *V* = 5000.
(PDF)

S4 FigLinearly unstable wave modes.Position of the peak of the dispersion relation (solid black line) versus the length of the filament Ω. The dashed and dotted lines denote the upper and lower bounds of the interval where the dispersion relation is positive. Notice that the wavenumber of the leading mode (solid line) grows linearly with the size of the filament Ω. Recalling that the spatial coordinate *x* appearing in Eq (39) of S1 Text is scaled by Ω, one can conclude that patterns on a growing domain present the same characteristic spacing as displayed on a fixed support. Parameters are set as *k*_*R*_ = 0.2, *α*_*R*_ = 0.2, *K* = 2, *k*_*S*_ = 0.1, *α*_*S*_ = 0.1, *μ*_*S*_ = 0.1, *k*_*N*_ = 0.7, *α*_*N*_ = 0.3, *μ*_*N*_ = 3, *D*_*S*_ = 3, *D*_*N*_ = 1, and $\tilde{\rho }=5\cdot 10^{-5}$.(PDF)

S1 Text Details on the mathematical calculations. (PDF)

S1 Data Experimental and numerical data used in figures. (XLS)

## Abbreviations

GFP: green fluorescent protein
Sm: streptomycin sulfate
Sp: spectinomycin dihydrochloride pentahydrate
