# Supplementary figures and images for: Robust stochastic Turing patterns in the development of a one-dimensional cyanobacterial organism
Source: PLoS Biol. 2018 May 4;16(5):e2004877. doi: 10.1371/journal.pbio.2004877 (PMC5955598; doi:10.1371/journal.pbio.2004877)

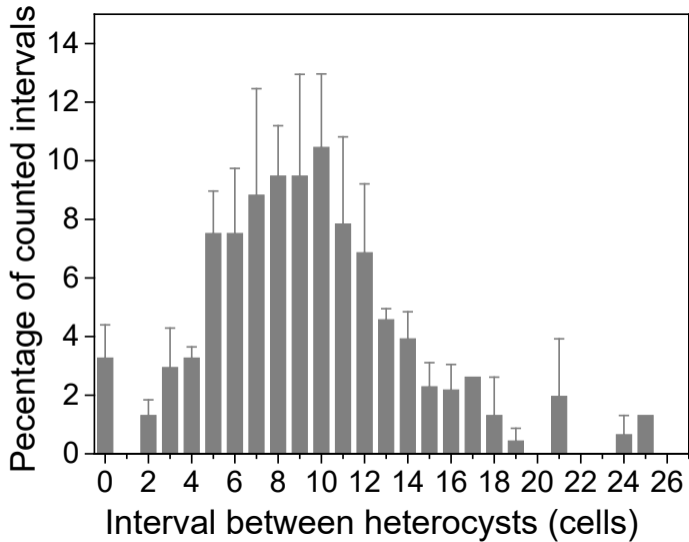

Supplement: S1 Fig — Filaments from ammonium-supplemented cultures were washed three times with BG110 medium, resuspended in BG110 medium, and grown in one of our devices prepared with BG110 medium (see Methods). Filaments were followed in real time to ascertain which cells became heterocysts. Intervals (n = 248) were counted 24 h after nitrogen deprivation. The mean interval size is 9.3±0.5 cells, and error bars represent standard errors from five independent experimental runs. The data used in this figure are included in S1 Data. (PDF) [file pbio.2004877.s001.pdf]

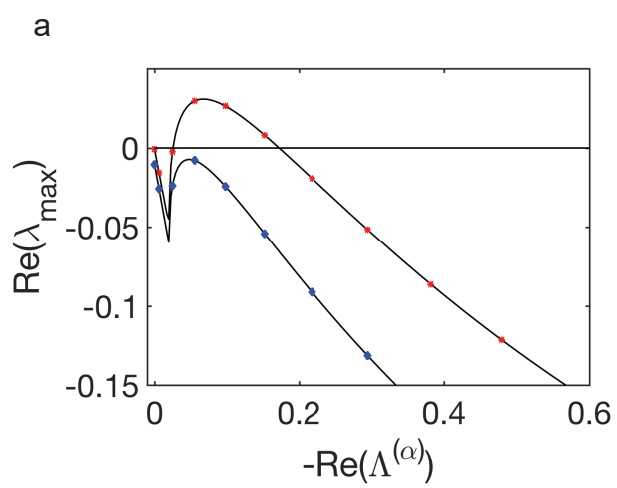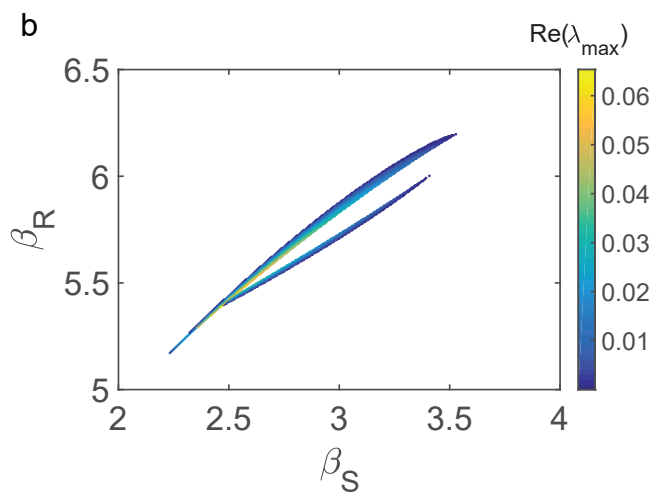

Supplement: S2 Fig — (a) Dispersion relations for βR = 5.69 and βS = 2.99 (blue diamonds) and βR = 5.82 and βS = 2.99 (red stars). The data used in this figure are included in S1 Data. (b) Region in the plane (βS,βR) where the maximum of λRe(Λ(α)) is positive, and the equilibrium point is stable for a ratio of diffusion coefficients DSDN=1. Parameters are set as kR = 0.2, αR = 0.2, K = 2, kS = 0.1, αS = 0.1, μS = 0.1, kN = 0.7, αN = 0.3, μN = 3, DS = 4, DN = 4, and Ω = 40. (PDF) [file pbio.2004877.s002.pdf]

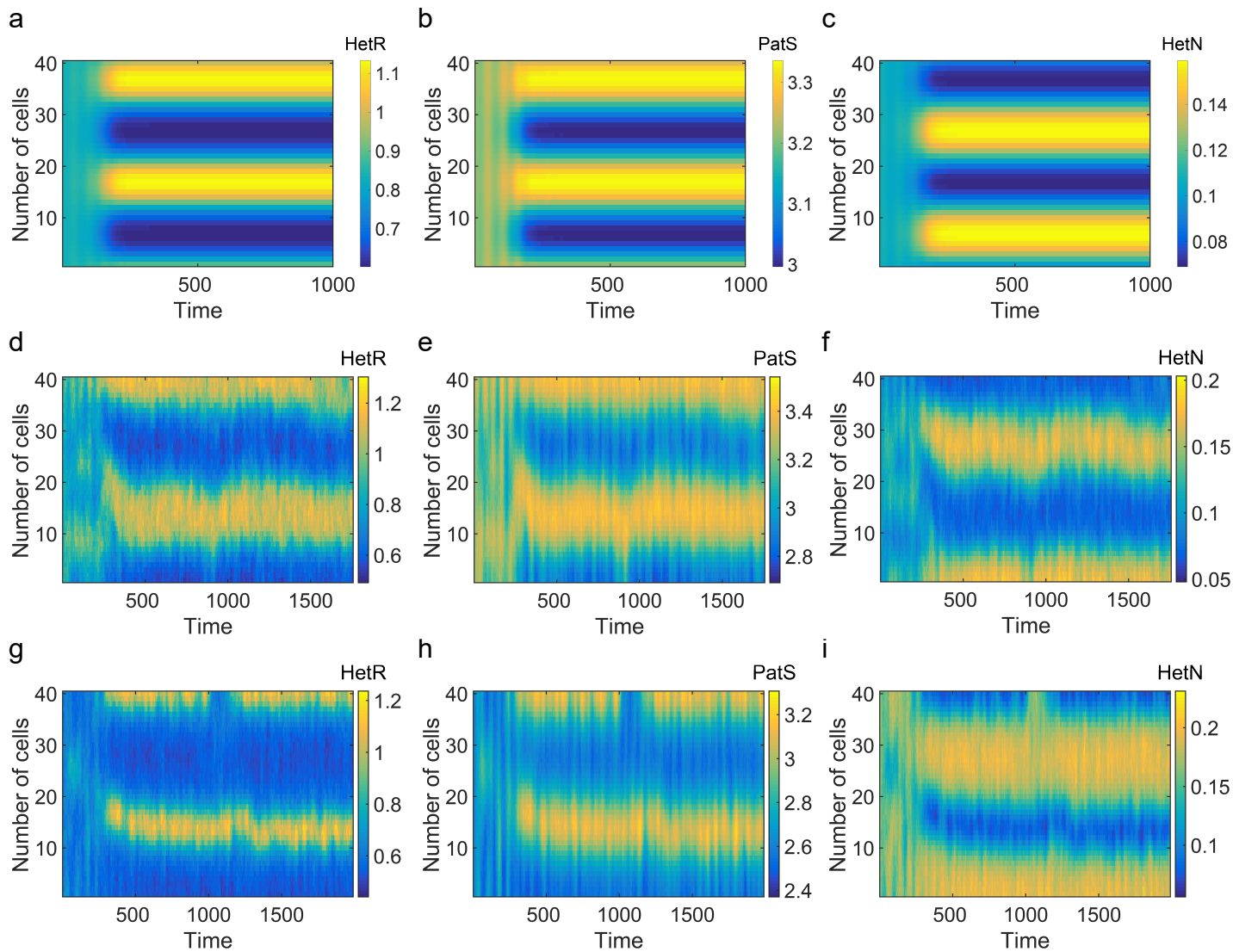

Supplement: S3 Fig — (a—c) Numerical integration of Eq (9) of the main text. (d–f) Stochastic simulations using the Gillespie algorithm. Parameters correspond to those used to compute the red stars curve of Fig 3A and apply to all panels from (a) to (f). (g–i) Stochastic Turing patterns corresponding to the blue diamonds curve of Fig 3A. For all panels, Ω = 40 and V = 5000. (PDF) [file pbio.2004877.s003.pdf]

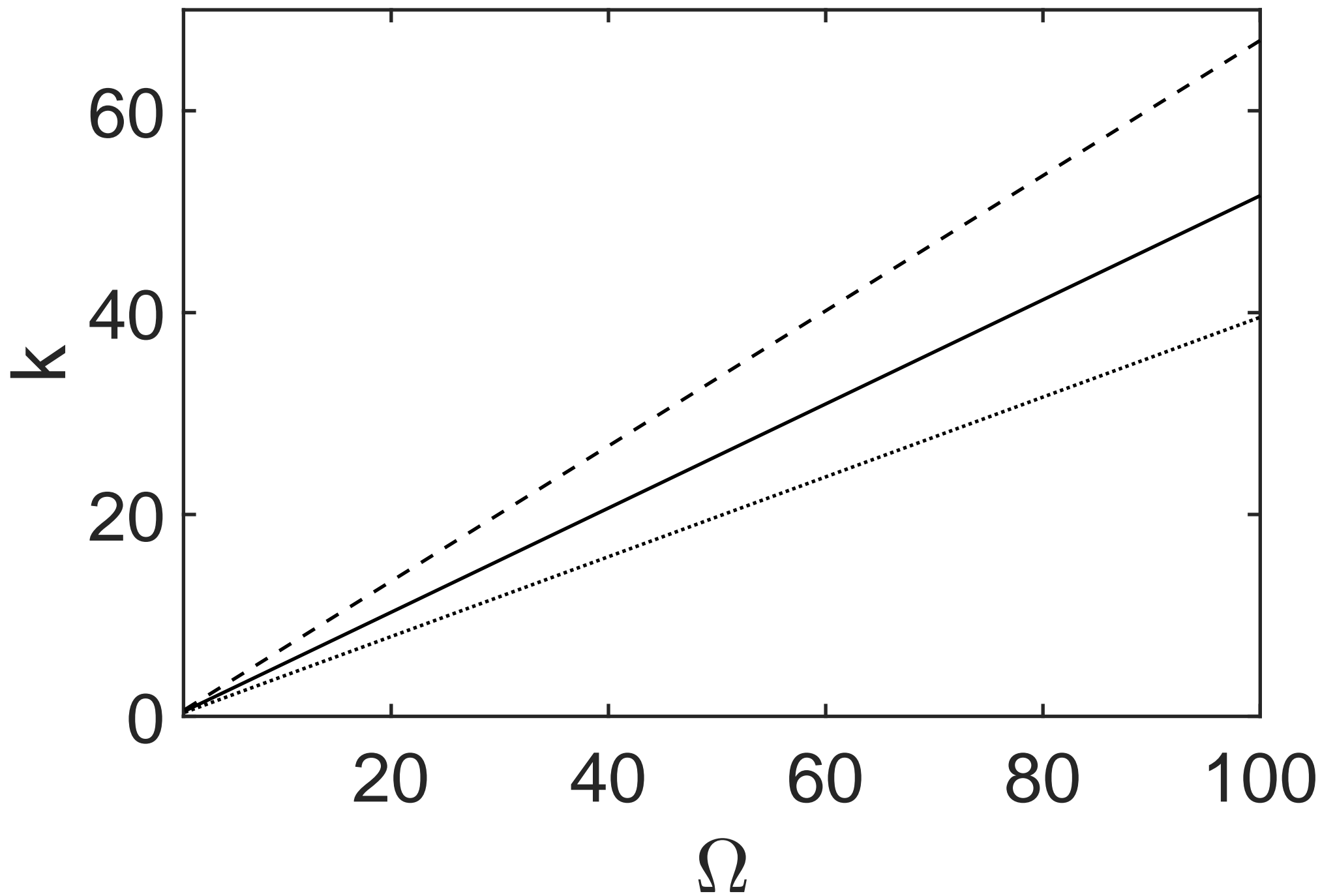

Supplement: S4 Fig — Position of the peak of the dispersion relation (solid black line) versus the length of the filament Ω. The dashed and dotted lines denote the upper and lower bounds of the interval where the dispersion relation is positive. Notice that the wavenumber of the leading mode (solid line) grows linearly with the size of the filament Ω. Recalling that the spatial coordinate x appearing in Eq (39) of S1 Text is scaled by Ω, one can conclude that patterns on a growing domain present the same characteristic spacing as displayed on a fixed support. Parameters are set as kR = 0.2, αR = 0.2, K = 2, kS = 0.1, αS = 0.1, μS = 0.1, kN = 0.7, αN = 0.3, μN = 3, DS = 3, DN = 1, and ρ˜=5⋅10−5. (PDF) [file pbio.2004877.s004.pdf]
