# Supplementary material for: Robust stochastic Turing patterns in the development of a one-dimensional cyanobacterial organism
Source: PLoS Biol. 2018 May 4;16(5):e2004877. doi: 10.1371/journal.pbio.2004877 (PMC5955598; doi:10.1371/journal.pbio.2004877)
Supplement: S1 Text — (PDF) [file pbio.2004877.s005.pdf]

Supporting text – Robust stochastic Turing patterns in the development of a one-dimensional cyanobacterial organism

Francesca Di Patti,[1,2] Laura Lavacchi,[1,3] Rinat Arbel-Goren,[4]
Leora Schein-Lubomirsky,[4] Duccio Fanelli,[1,2] and Joel Stavans[4]
[1] *Università degli Studi di Firenze, Dipartimento di Fisica e Astronomia, via G. Sansone 1, 50019 Sesto Fiorentino, Italia*
[2] *CSDC and INFN Sez.di Firenze, via G. Sansone 1, 50019 Sesto Fiorentino, Italia*
[3] *Department of Physics, Freie Universitat Berlin, Arnimalee 14, 14195 Berlin, Germany*
[4] *Department of Physics of Complex Systems, Weizmann Institute of Science, Rehovot 76100, Israel*

I. *ANABAENA* GROWTH AND DEVELOPMENT WITHIN DEVICES

To test whether *Anabaena* growth and developmental features are altered when filaments are grown within devices under our microscope, we measured the doubling time of vegetative cells under nitrogen-rich conditions. We obtained 17.4 hr, which compares favorably with values measured in the bulk [3]. In addition, the heterocyst spacing distributions we observe 24 h following nitrogen step-down (S1 Fig) are consistent with those obtained in the bulk in a wild-type background (see e.g. [21] in the paper). Lastly, we note that the onset of HetN-GFP expression takes place *circa* 16 h after nitrogen step-down, consistently with previous experimental observations [5]. Thus under our experimental conditions, filament growth and development within devices are similar to those in bulk cultures.

II. DETAILS ON THE STOCHASTIC MODEL

We begin this section by listing all the transitions rates that need to be accommodated for in the master equation for the stochastic model on a static domain:

$$
\begin{aligned}
T_1(r_i+1|r_i) &= \alpha_R \\
T_2(r_i-1|r_i) &= k_R \frac{r_i}{V} \\
T_3(r_i+1|r_i) &= \beta_R \frac{(\frac{r_i}{V})^2}{K^2+(\frac{r_i}{V})^2} \\
T_4(r_i-2, s_i-1|r_i, s_i) &= \mu_S \left(\frac{r_i}{V}\right)^2 \frac{s_i}{V} \\
T_5(r_i-2, n_i-1|r_i, n_i) &= \mu_N \left(\frac{r_i}{V}\right)^2 \frac{n_i}{V} \\
T_6(s_i+1|s_i) &= \alpha_S \\
T_7(s_i-1|s_i) &= k_S \frac{s_i}{V} \\
T_8(s_i+1|s_i) &= \beta_S \frac{(\frac{r_i}{V})^2}{K^2+(\frac{r_i}{V})^2} \\
T_9(n_i+1|n_i) &= \alpha_N \\
T_{10}(n_i-1|n_i) &= k_N \frac{n_i}{V} \\
T_{11}(s_i-1, s_j+1|s_i, s_j) &= D_S \frac{s_i}{V} \\
T_{12}(n_i-1, n_j+1|n_i, n_j) &= D_N \frac{n_i}{V}
\end{aligned}
\tag{1}
$$

The above transitions rates stem from the chemical equations displayed, and thoroughly discussed, in the main body of the paper.

The governing master equation can be written in the compact form

$$\begin{aligned}
&\frac{d}{dt}P(\mathbf{r},\mathbf{s},\mathbf{n},t) = \\
&\sum_i^N \Big[(\epsilon_{1,i}^{-}-1)(T_1(r_i+1|r_i)+T_3(r_i+1|r_i))+ \\
&+(\epsilon_{1,i}^{+}-1)T_2(r_i-1|r_i)+ \\
&+(\epsilon_{2,i}^{-}-1)\Big(T_6(s_i+1|s_i)+T_8(s_i+1|s_i)\Big)+ \\
&+(\epsilon_{2,i}^{+}-1)T_7(s_i-1|s_i)+ \\
&+(\epsilon_{3,i}^{-}-1)T_9(n_i+1|n_i)+ \\
&+(\epsilon_{3,i}^{+}-1)T_{10}(n_i-1|n_i)+ \\
&+(\epsilon_{1,i}^{+}\epsilon_{1,i}^{+}\epsilon_{2,i}^{+}-1)T_4(r_i-2,s_i-1|r_i,s_i)+ \\
&+(\epsilon_{1,i}^{+}\epsilon_{1,i}^{+}\epsilon_{2,i}^{+}-1)T_5(r_i-2,n_i-1|r_i,n_i)+ \\
&+\sum_j^N W_{ij}[(\epsilon_{2,i}^{+}\epsilon_{2,j}^{-}-1)T_{11}(s_i-1,s_j+1|s_i,s_j)+ \\
&+(\epsilon_{3,i}^{+}\epsilon_{3,j}^{-}-1)T_{12}(n_i-1,n_j+1|n_i,n_j)]\Big]P(\mathbf{r},\mathbf{s},\mathbf{n},t)
\end{aligned} \tag{2}$$

where use has been made of the so-called step operators. The step operators act on a generic function f as specified by:

$$\begin{aligned}
\epsilon_{1,i}^{\pm}f(\mathbf{r},\mathbf{s},\mathbf{n}) &= f(\dots,r_i\pm 1,\dots,\mathbf{s},\mathbf{n}) \\
\epsilon_{2,i}^{\pm}f(\mathbf{r},\mathbf{s},\mathbf{n}) &= f(\mathbf{r},\dots,s_i\pm 1,\dots,\mathbf{n}) \\
\epsilon_{3,i}^{\pm}f(\mathbf{r},\mathbf{s},\mathbf{n}) &= f(\mathbf{r},\mathbf{s},\dots,n_i\pm 1,\dots)
\end{aligned}$$

The scalar quantities W_{ij} are the entries of the adjacency matrix $\mathbf{W}$ that defines the spatial structure of the model. As explained in the main body of the paper, the *Anabaena* filament can be modeled as a one-dimensional lattice, with non-directional nearest-neighbors couplings. The lattice is open and so the first and last cells of the chain are connected to just one adjacent neighbor. With an obvious meaning of the symbols involved we therefore posit:

$$\mathbf{W} = \begin{pmatrix} 0 & 1 & \dots & 0 & 0 \\ 1 & 0 & 1 & \dots & 0 \\ \vdots & \vdots & \vdots & \vdots & \vdots \\ \dots & \dots & 1 & 0 & 1 \\ 0 & 0 & \dots & 1 & 0 \end{pmatrix} \tag{3}$$

III. DETAILS ON THE VAN KAMPEN EXPANSION

As explained in the main body of the paper the van Kampen expansion amounts to split the stochastic density of the regulators into two parts: the first represents the mean-field concentration, as recovered in the thermodynamic limit. The second qualifies as a stochastic contribution scaled by the amplitude factor $1/\sqrt{V}$, as dictated by central limit theorem. For moderate system sizes (i.e. working at finite V), the quantity $1/\sqrt{V}$ acts as a perturbative parameter in the expansion technique, pioneered by van Kampen. In the following we provide some key steps to reproduce the calculations that yield the formulae reported in the main body of the paper.

From the van Kampen ansatz, the left-hand side of the master equation can be written as:

$$\frac{d}{dt}P(\mathbf{r},\mathbf{s},\mathbf{n},t) = \sum_i^{\Omega}\left(\frac{\partial\Pi}{\partial t} - \frac{\partial\Pi}{\partial\xi_{1,i}}\sqrt{V}\dot{\phi}_i - \frac{\partial\Pi}{\partial\xi_{2,i}}\sqrt{V}\dot{\psi}_i - \frac{\partial\Pi}{\partial\xi_{3,i}}\sqrt{V}\dot{\eta}_i\right) \tag{4}$$

where $\Pi(\xi_{\mathbf{1}},\xi_{\mathbf{2}},\xi_{\mathbf{3}},t) \equiv P(\mathbf{r},\mathbf{s},\mathbf{n},t)$ represents the probability distribution function of fluctuations.

Then, the step operators (and their combinations as appearing in the master equation) can be expanded as follows:

$$\epsilon_{1,i}^{\pm} \simeq 1 \pm \frac{1}{\sqrt{V}}\frac{\partial}{\partial_{\xi_{2,i}}} + \frac{1}{2V}\frac{\partial^2}{\partial_{\xi^2_{3,i}}} \tag{5}$$

$$(\epsilon_{1,i}^{+}\epsilon_{1,j}^{-} - 1) \simeq \frac{1}{\sqrt{V}}\Big(\frac{\partial}{\partial_{\xi_{1,i}}} - \frac{\partial}{\partial_{\xi_{1,j}}}\Big) + \frac{1}{2V}\Big(\frac{\partial^2}{\partial_{\xi^2_{1,i}}} + \frac{\partial^2}{\partial_{\xi^2_{1,j}}} - 2\frac{\partial}{\partial_{\xi_{1,i}}}\frac{\partial}{\partial_{\xi_{1,j}}}\Big) \tag{6}$$

$$(\epsilon_{1,i}^{+}\epsilon_{1,i}^{+}\epsilon_{2,i}^{+} - 1) \simeq \frac{1}{\sqrt{V}}\Big(\frac{\partial}{\partial_{\xi_{2,i}}} + 2\frac{\partial}{\partial_{\xi_{1,i}}}\Big) + \frac{1}{2V}\Big(\frac{\partial^2}{\partial_{\xi^2_{2,i}}} + 4\frac{\partial^2}{\partial_{\xi^2_{1,i}}} + 4\frac{\partial}{\partial_{\xi_{1,i}}}\frac{\partial}{\partial_{\xi_{2,i}}}\Big) \tag{7}$$

The above (and other homologous) expressions can be inserted in the right hand side of the master equation. Similarly, one can proceed by expanding the transition rates (details not provided), upon introduction of the van Kampen ansatz. Scaling time as $\tau = t/V$ and collecting the leading terms in the expansion, yields the deterministic mean field equations for the three coupled species, as reported in the main body of the paper.

At the next to leading order, one obtains a Fokker-Planck equation for the evolution of the distribution Π, namely:

$$\frac{\partial}{\partial \tau}\Pi = \sum_{i}^{\Omega}\Bigg(-\sum_{q=1}^{3}\frac{\partial}{\partial \xi_{q,i}}(A_{q,i}\Pi) + \frac{1}{2}\sum_{q,l=1}^{3}\sum_{j}^{\Omega}(B_{ql,ij}\Pi)\Bigg) \tag{8}$$

with

$$A_{q,i} = \sum_{l=1}^{3}\sum_{j}^{\Omega} M_{rl,ij}\xi_{s,j} \tag{9}$$

and where the size of matrices $\mathbf{M}$ and $\mathbf{B}$ is $3\Omega \times 3\Omega$. In the following we will assume the above operators to be evaluated at the fixed point (ϕ^*, ψ^*, η^*).

Matrix $\mathbf{M}$ can be split into two terms, one retaining the spatial contributions ($M^{(SP)}$) and one with contributions that come from the reaction terms ($M^{(NS)}$). In formulae:

$$M_{ql,ij} = M_{ql}^{(NS)}\delta_{ij} + M_{ql}^{(SP)}\Delta_{ij} \tag{10}$$

where:

$$\begin{aligned}
M_{11}^{(NS)} &= -\Bigg(k_R - 2\frac{\beta_R\phi^*}{K^2+(\phi^*)^2}\Big(1 - \frac{(\phi^*)^2}{K^2+(\phi^*)^2}\Big) + 4\mu_S\phi^*\psi^* + 4\mu_N\phi^*\eta^*\Bigg)\\
M_{12}^{(NS)} &= -2\mu_S(\phi^*)^2\\
M_{13}^{(NS)} &= -2\mu_N(\phi^*)^2\\
M_{21}^{(NS)} &= 2\frac{\beta_S\phi^*}{K^2+(\phi^*)^2}\Big(1 - \frac{(\phi^*)^2}{K^2+(\phi^*)^2}\Big) - 2\mu_S\phi^*\psi^*\\
M_{22}^{(NS)} &= -k_S - \mu_S(\phi^*)^2\\
M_{23}^{(NS)} &= 0\\
M_{31}^{(NS)} &= -2\mu_N\phi^*\eta^*\\
M_{32}^{(NS)} &= 0\\
M_{33}^{(NS)} &= -k_N - \mu_N(\phi^*)^2
\end{aligned} \tag{11}$$

while the entries of $M^{(SP)}$ are zeros except those corresponding to the mobile species, namely

$$\begin{aligned}
M_{22}^{(SP)} &= D_S\\
M_{33}^{(SP)} &= D_N
\end{aligned} \tag{12}$$

Analogously, B can be split into spatial and non-spatial components as:

$$B_{ql,ij} = B_{ql}^{(NS)}\delta_{ij} + B_{ql}^{(SP)}\Delta_{ij} \tag{13}$$

where

$$\begin{aligned}
B_{11}^{(NS)} &= \alpha_R + k_R\phi^* + \beta_R\frac{(\phi^*)^2}{K^2+(\phi^*)^2} + 4\mu_S(\phi^*)^2\psi^* + 4\mu_N(\phi^*)^2\eta^* && (14)\\
B_{12}^{(NS)} &= 2\mu_S(\phi^*)^2\psi^* && (15)\\
B_{13}^{(NS)} &= 2\mu_N(\phi^*)^2\eta_i && (16)\\
B_{21}^{(NS)} &= 2\mu_S(\phi^*)^2\psi^* && (17)\\
B_{22}^{(NS)} &= \alpha_S + k_S\psi^* + \beta_S\frac{(\phi^*)^2}{K^2+(\phi^*)^2} + \mu_S(\phi^*)^2\psi^* && (18)\\
B_{23}^{(NS)} &= 0 && (19)\\
B_{31}^{(NS)} &= 2\mu_N(\phi^*)^2\eta^* && (20)\\
B_{32}^{(NS)} &= 0 && (21)\\
B_{33}^{(NS)} &= \alpha_N + k_N\eta^* + \mu_N(\phi^*)^2\eta^* && (22)
\end{aligned}$$

and the entries of $B^{(SP)}$ are zeros except

$$\begin{aligned}
B_{22}^{(SP)} &= -2D_S\psi^* \\
B_{33}^{(SP)} &= -2D_N\eta^* \quad .
\end{aligned} \tag{23}$$

IV. EQUILIBRIUM POINTS

To calculate the homogeneous fixed point of the deterministic model, we look for solutions ($\dot{\phi}_i = \dot{\psi}_i = \dot{\eta}_i = 0$) such that $\phi_i = \phi^*$, $\psi_i = \psi^*$, $\eta_i = \eta^*$ for all i.

An immediate manipulation of the reference equations yields:

$$\begin{cases}
\psi^* = \dfrac{1}{k_S + \mu_S(\phi^*)^2}\left(\alpha_S + \beta_S\dfrac{(\phi^*)^2}{K^2+(\phi^*)^2}\right) \\[2ex]
\eta^* = \dfrac{\alpha_N}{k_N + \mu_N(\phi^*)^2}
\end{cases} \tag{24}$$

and, by inserting (24) into the first equation of the deterministic system reported in the main body of the paper, one ends up with the following seventh-order polynomial:

$$c_7(\phi^*)^7 + c_6(\phi^*)^6 + c_5(\phi^*)^5 + c_4(\phi^*)^4 + c_3(\phi^*)^3 + c_2(\phi^*)^2 + c_1(\phi^*) + c_0 = 0 \tag{25}$$

with coefficients

$$\begin{aligned}
c_7 &= -k_R \\
c_6 &= \alpha_R + \beta_R - 2\alpha_S - 2\beta_S - 2\alpha_N \\
c_5 &= -k_R(K^2 + t_S + t_N) \\
c_4 &= \alpha_R(K^2 + t_S + t_N) + \beta_R(t_N + t_S) - 2\alpha_S(t_N + K^2) - 2\beta_S t_N - 2\alpha_S(t_N + K^2) \\
c_3 &= -k_R(K^2(t_S + t_N) + t_S t_N) \\
c_2 &= \alpha_R(K^2(t_S + t_N) + t_S t_N) + \beta_R t_S t_N - 2\alpha_S t_N K^2 - 2\alpha_N t_S K^2 \\
c_1 &= -k_R K^2 t_S t_N \\
c_0 &= \alpha_R t_S t_N K^2
\end{aligned} \tag{26}$$

where $t_S = \frac{k_S}{\mu_S}$ and $t_N = \frac{k_N}{\mu_N}$. The real and positive roots of the above polynomial return the sought homogeneous equilibria. The stability of the homogeneous solutions is then determined by computing the eigenvalues of the following Jacobian matrix:

$$J = \begin{pmatrix} F & -\mu_S(\phi^*)^2 & -\mu_N(\phi^*)^2 \\ G & -k_S - \mu_S(\phi^*)^2 & 0 \\ -2\mu_N\phi^*\eta^* & 0 & -k_N - \mu_N(\phi^*)^2 \end{pmatrix} \tag{27}$$

with $F = -k_R + \beta_R \frac{2\phi^* K^2}{(K^2+(\phi^*)^2)^2} - 2\mu_S\phi^*\psi^* - 2\mu_N\phi^*\eta^*$ end $G = \beta_S \frac{2\phi^* K^2}{(K^2+(\phi^*)^2)^2} - 2\mu_S\phi^*\psi^*$.

V. TURING INSTABILITY CONDITIONS FOR A THREE-SPECIES MODEL

This section is aimed at elaborating on the conditions that underlie the Turing instability for a three species model defined on a one-dimensional support. We will in particular reproduce a selection of the results reported in [1], where this generalization is thoroughly discussed.

As discussed in the main body of the paper, the conditions for the instability of a reaction diffusion system defined on a discrete (symmetric) support can be derived by operating in the limit of a continuum spatial medium, i.e. by replacing the discrete Laplacian with its continuum counterpart. In the following we shall assume, as in [1], that the three species are characterized in terms of their concentrations $H(x,t), P(x,t), Q(x,t)$: the species can undergo generic reactions with each other and relocate in space, as follows standard diffusion. This translates into the following formulae:

$$\begin{cases} \frac{\partial H}{\partial t} = A(H,P,Q) + D_H \frac{\partial^2 H}{\partial x^2} \\ \\ \frac{\partial P}{\partial t} = B(H,P,Q) + D_P \frac{\partial^2 P}{\partial x^2} \\ \\ \frac{\partial Q}{\partial t} = C(H,P,Q) + D_Q \frac{\partial^2 Q}{\partial x^2} \end{cases} \tag{28}$$

where $A(H,P,Q), B(H,P,Q), C(H,P,Q)$ are non-linear functions denoting the reaction terms. The homogeneous equilibrium point (H^*, P^*, Q^*) satisfies

$$\begin{cases} A(H^*, P^*, Q^*) = 0 \\ B(H^*, P^*, Q^*) = 0 \\ C(H^*, P^*, Q^*) = 0 \end{cases}$$

We introduce small non homogeneous perturbations to the steady state (H^*, P^*, Q^*):

$$\begin{cases} H(x,t) = H^* + h(x,t) \\ P(x,t) = P^* + p(x,t) \\ Q(x,t) = Q^* + q(x,t) \end{cases} \tag{29}$$

and choose the perturbations in the form

$$\begin{cases} h(x,t) = h_0 e^{\lambda t + ikx} \\ p(x,t) = p_0 e^{\lambda t + ikx} \\ q(x,t) = q_0 e^{\lambda t + ikx} \end{cases} \tag{30}$$

where h_0, p_0 and q_0 are constant parameters and k stands for the continuum wavenumber.

Inserting (29) and (30) into (28), after some algebraic manipulations, one ends up with the following condition

$$\begin{vmatrix} \lambda - A_H + D_H k^2 & -A_P & -A_Q \\ -B_H & \lambda - B_P + D_P k^2 & -B_Q \\ -C_H & -C_P & \lambda - C_Q + D_Q k^2 \end{vmatrix} = 0 \tag{31}$$

where we have adopted the notation $A_H = \frac{\partial A}{\partial H} |_{(H^*,P^*,Q^*)}$ (the same for the other species). The dispersion relation becomes

$$\lambda^3 + d_1(k^2)\lambda^2 + d_2(k^2)\lambda + d_3(k^2) = 0 \tag{32}$$

with

$$
\begin{aligned}
d_1(k^2) =& - A_H - C_Q - B_P + k^2(D_H + D_P + D_Q) \\
d_2(k^2) =& A_H C_Q + A_H B_P + B_P C_Q - C_P B_Q - A_P B_H - A_Q C_H \\
& - k^2(D_Q B_P + D_P C_Q + D_H C_Q + D_H B_P + D_P A_H + D_Q A_H) \\
& + k^4(D_P D_Q + D_P D_H + D_H D_Q) \\
d_3(k^2) =& - A_H B_P C_Q + A_H C_P B_Q + A_P B_H C_Q + \\
& - A_P C_H B_Q - A_Q B_H C_P + C_H B_P A_Q \\
& + k^2(-D_Q A_P B_H - D_P C_H A_Q - D_H C_P B_Q + \\
& + D_H B_P C_Q + D_P C_Q A_H + D_Q B_P A_H) \\
& - k^4(D_P D_H C_Q + D_H D_Q B_P + D_P D_Q A_H) \\
& + k^6 D_H D_P D_Q
\end{aligned}
\tag{33}
$$

The conditions for the steady states to be stable ($\text{Re}(\lambda) < 0$), implies:

$$
d_1(k^2) > 0, \quad d_3(k^2) > 0, \quad d_1(k^2)d_2(k^2) - d_3(k^2) > 0 \tag{34}
$$

Since in our model only two species diffuse, we set to zero the diffusion coefficient of the first species, namely $D_H = 0$. In this way, $d_3(k^2)$ becomes a quadratic function of k^2, namely:

$$
d_3(k^2) = -D_P D_Q A_H k^4 + k^2[D_P(C_Q A_H - C_H A_Q) + D_Q(B_P A_H - A_P B_H)] + d_3(0) \tag{35}
$$

If $d_3(k^2) < 0$, the aforementioned conditions (34) are violated and the system can turn unstable. This necessary condition can be further analyzed in terms of A_H. In particular, following the analysis reported in [1], one finds:

- If $A_H > 0, \quad d_3(k^2) \to -\infty$ if $k^2 \to +\infty$. No bounded domain in k^2 exists, where the instability is localized.
- If $A_H = 0$, there exist infinite values of k^2 which let the system unstable
- If $A_H < 0$, $d_3(k^2) < 0$ over a bounded domain in k^2, if and only if:

$$
\begin{aligned}
F_1 =& D_P(C_Q A_H - C_H A_Q) + D_Q(B_P A_H - A_P B_H) < 0 \\
F_2 =& [D_P(C_Q A_H - C_H A_Q) + D_Q(B_P A_H - A_P B_H)]^2 + 4 D_P D_Q A_H d_3(0) > 0
\end{aligned}
\tag{36}
$$

Summarizing, for a model with three species, two diffusing and one immobile, the deterministic Turing instability is possible if A_H is negative and constrains (36) are satisfied.

VI. DETAILS ON THE MODEL WITH GROWTH: THE MEAN FIELD DYNAMICS

Starting from the master equation modified so to account for the growth of the filament, one can obtain, via a straightforward manipulation, the mean field equations that govern the evolution of the system in the deterministic limit. These are the following $3\Omega(t) \times 3\Omega(t)$ differential equations [2]:

$$
\begin{cases}
\dot{\phi}_i &= \alpha_R - k_R \phi_i + \beta_R \frac{\phi_i^2}{K^2 + \phi_i^2} - 2\mu_S \phi_i^2 \psi_i - 2\mu_N \phi_i^2 \eta_i + \tilde{\rho}\Big[\big(i - \tfrac{3}{2}\big)\phi_{i-1} - \big(i - \tfrac{1}{2}\big)\phi_i\Big] \\
\dot{\psi}_i &= \alpha_S - k_S \psi_i + \beta_S \frac{\phi_i^2}{K^2 + \phi_i^2} - \mu_S \phi_i^2 \psi_i + D_S \sum_j^{\Omega} \Delta_{ij} \psi_i + \tilde{\rho}\Big[\big(i - \tfrac{3}{2}\big)\psi_{i-1} - \big(i - \tfrac{1}{2}\big)\psi_i\Big] \\
\dot{\eta}_i &= \alpha_N - k_N \eta_i(L) - \mu_N \phi_i^2 \eta_i + D_N \sum_j^{\Omega} \Delta_{ij} \eta_i(L) + \tilde{\rho}\Big[\big(i - \tfrac{3}{2}\big)\eta_{i-1} - \big(i - \tfrac{1}{2}\big)\eta_i\Big]
\end{cases}
\tag{37}
$$

where $\tilde{\rho} = V\rho$.

Taking the spatial continuum limit returns:

$$
\begin{cases}
\dot{\phi} = \alpha_R - k_R \phi + \beta_R \dfrac{\phi^2}{K^2 + \phi^2} - 2\mu_S \phi^2 \psi - 2\mu_N \phi^2 \eta - \tilde{\rho}\Big[x \dfrac{\partial \phi}{\partial x} + \phi\Big] \\
\dot{\psi} = \alpha_S - k_S \psi + \beta_S \dfrac{\phi^2}{K^2 + \phi^2} - \mu_S \phi^2 \psi + D_S \dfrac{\partial^2}{\partial x^2}\psi - \tilde{\rho}\Big[x \dfrac{\partial \psi}{\partial x} + \psi\Big] \\
\dot{\eta} = \alpha_N - k_N \eta - \mu_N \phi^2 \eta + D_N \dfrac{\partial^2}{\partial x^2}\eta - \tilde{\rho}\Big[x \dfrac{\partial \eta}{\partial x} + \eta\Big]
\end{cases}
\tag{38}
$$

where now $\phi = \phi(x,t)$, $\psi = \psi(x,t)$ and $\eta = \eta(x,t)$. Moreover $D_{S,N} = \lim_{\Delta x \to 0} \Delta x^2 D_{S,N}$, Δx labeling the linear size of the discrete mesh. The effect of the growth can be formally scaled out by performing the following change of variables $(x,\tau) \to (\tilde{x},\tilde{\tau}) = \left(\frac{x}{\Omega(\tau)},\tau\right)$. Here, $\Omega(\tau) = \Omega_0 \exp(\tilde{\rho}\tau)$ and Ω_0 stands for the number of cells that compose the filament at $\tau = 0$.

An immediate calculation yields:

$$\begin{cases} \dot{\phi} = \alpha_R - k_R\phi + \beta_R \dfrac{\phi^2}{K^2+\phi^2} - 2\mu_S\phi^2\psi - 2\mu_N\phi^2\eta - \tilde{\rho}\phi \\ \dot{\psi} = \alpha_S - k_S\psi + \beta_S \dfrac{\phi^2}{K^2+\phi^2} - \mu_S\phi^2\psi + \dfrac{D_S}{\Omega(t)^2}\dfrac{\partial^2}{\partial x^2}\psi - \tilde{\rho}\psi \\ \dot{\eta} = \alpha_N - k_N\eta - \mu_N\phi^2\eta + \dfrac{D_N}{\Omega(t)^2}\dfrac{\partial^2}{\partial x^2}\eta - \tilde{\rho}\eta \end{cases} \tag{39}$$

where, for the ease of notation, we consistently dropped, in the above equations, the tilde sign in $(\tilde{x},\tilde{\tau})$. The growth materializes in the rescaled equations as (i) linear dilution terms, which affect all involved species, and (ii) time dependent diffusion coefficients. System (39) can be readily simulated and the results displayed back in the original spatial support so to make the effect of the growth visible. Reflecting boundary conditions are assumed at the edges.

VII. SIMULATIONS WITH $D_S/D_N = 1$

Endogenous demographic noise makes the region of Turing instability larger, and hence facilitates the process of pattern formation in *Anabaena*. This contribution is particularly relevant when $D_S/D_N = 1$. In this case, in fact, the domain of parameters that promotes the instability is very small (see S2 Fig panel b). Noise enlarges the domain of deterministic instability, yielding robust patterns also for a choice of the parameters that would return a stable homogeneous fixed point in the mean field approximation (see S3 Fig panels g-i). Notice that parameters could be in principle optimized so as to return the spacing between heterocysts, as displayed in real experiments.

VIII. LINEARLY GROWING WAVE MODES

The growth of the filament increases the size of the domain in k that corresponds to a positive dispersion relation (see S4 Fig). For a given length Ω, we have applied the Turing instability analysis to system (39) and recorded the position of the maximum of the dispersion relation (solid line). The dashed and dotted lines identify the values of k that yield a dispersion relation equal to zero. In other words, the dashed and dotted lines delimit the interval in k where the modes triggered unstable are located.

[1] K.A. White and C.A. Gillian, Phil. Trans. R. Soc. Lond. B **353** 543-555 (1998).
[2] R. Baker, C. A. Yates, R. Erhon, Bulletin of mathematical biology, **72** (3), 719-762 (2010).
[3] J.E. Frias, E. Flores and A. Herrero, J. Bact., **179**, 477-486 (1997).
[4] T.A. Black and C.P. Wolk, J. Bacteriol. **176**, 2282-2292 (1994)
[5] S.M. Callahan and W.J. Buikema, Mol. Microbiol. **40**, 941-950 (2001).
